# Farm management practices, biosecurity and influenza a virus detection in swine farms: a comprehensive study in Colombia

**DOI:** 10.1186/s40813-022-00287-6

**Published:** 2022-10-05

**Authors:** Karl Ciuoderis-Aponte, Andres Diaz, Carlos Muskus, Mario Peña, Juan Hernández-Ortiz, Jorge Osorio

**Affiliations:** 1 Universidad Nacional de Colombia sede Medellín. Consortium Colombia Wisconsin One Health, Cra 75#61-85, 050034 Medellín, Colombia; 2 Pig Improvement Company, Querato, Mexico; 3 Programa de Estudio y Control de Enfermedades Tropicales- PECET, Universidad de Antioquia, Medellín, Colombia; 4 Asociación Porkcolombia - Fondo nacional de la porcicultura, Bogotá, Colombia; 5 Department of Pathobiological sciences, University of Wisconsin-Madison. Consortium Colombia Wisconsin One Health, 53706 Madison, USA

**Keywords:** Swine biosecurity, Husbandry practices, Swine farms, Multiple correspondence analysis, Hierarchical cluster analysis, Bayesian generalized linear model, Epidemiology, Swine influenza

## Abstract

Biosecurity protocols (BP) and good management practices are key to reduce the risk of introduction and transmission of infectious diseases into the pig farms. In this observational cross-sectional study, survey data were collected from 176 pig farms with inventories over 100 sows in Colombia. We analyzed a complex survey dataset to explore the structure and identify clustering patterns using Multiple Correspondence Analysis (MCA) of swine farms in Colombia, and estimated its association with Influenza A virus detection. Two principal dimensions contributed to 27.6% of the dataset variation. Farms with highest contribution to dimension 1 were larger farrow-to-finish farms, using self-replacement of gilts and implementing most of the measures evaluated. In contrast, farms with highest contribution to dimension 2 were medium to large farrow-to-finish farms, but implemented biosecurity in a lower degree. Additionally, two farm clusters were identified by Hierarchical Cluster Analysis (HCA), and the odds of influenza A virus detection was statistically different between clusters (OR 7.29, CI: 1.7,66, p = < 0.01). Moreover, after logistic regression analysis, three important variables were associated with higher odds of influenza detection: (1) “location in an area with a high density of pigs”, (2) “farm size”, and (3) “after cleaning and disinfecting, the facilities are allowed to dry before use”. Our results revealed two clustering patterns of swine farms. This systematic analysis of complex survey data identified relationships between biosecurity, husbandry practices and influenza status. This approach helped to identify gaps on biosecurity and key elements for designing successful strategies to prevent and control swine respiratory diseases in the swine industry.

**Supplementary Information:**

The online version contains supplementary material available at 10.1186/s40813-022-00287-6.

## Introduction

Influenza A viruses (IAV) are significant pathogens of humans, livestock and several wild species. They have a complex epidemiology due to their ability to cross species barriers [1]. These viruses are considered endemic in swine populations [2]. Swine Influenza (SI) is the most prevalent respiratory disease in pig productions worldwide [3]. As in humans, IAV epidemics in swine are generally associated with high morbidity and low mortality [7]. Increase on feed conversion, weight loss and reduce average daily gain of pigs are the main negative effects of SI, which significantly affects the productivity of growing pigs and the reproductive performance of breeding sows [4–7]. Some studies have estimated a global and herd-level seroprevalence in pigs of 49.9 and 72.8% respectively [8]. The first report of IAV in Colombia dated from the 70’s [9]; while pandemic and classical H1 virus were detected during the last decade [10]. However, scarce information is available regarding IAV epidemiology and prevalence in pig farms in this country.

Additionally, SI is usually observed as a component of Porcine Respiratory Disease Complex, which significatively influence the profitability of a farm [11]. Also, SI is associated with higher mortality rates of piglets [12] and increased cost of production [13]. Furthermore, the 2009 pandemic provided an increased concern of the potential damage to the pork industry of indirect costs that are incurred from IAV zoonotic transmission [14]. The pig plays an important role in IAV ecology because of its ability to support replication of viruses from avian, swine and human origins [15]. To date, the epidemiology of SI is not fully understood and multiple factors affecting SI infections have been described [16]. Furthermore, the dynamic genome of IAV and the complexity of molecular epidemiology in sow herds [17] and in pigs after weaning [18] increases the challenges of understanding and controlling SI in pig farms. Transmission of SI in pigs can occur via direct pig-to-pig contact, exposure to fomites and aerosols although other indirect routes of transmission are reported [15]. There is no effective treatment of SI and vaccination is currently one of the main tools to limit the disease transmission in swine [19]. Diversity of IAV can affect and reduce the vaccines efficacy in the field [13]. However, under the right conditions, SI vaccines can reduce or eliminate transmission of IAV in pig herds and thus their optimal use and effectivity would rely on information gathered from diagnostics and surveillance programs [13, 20–22]. On the other hand, SI vaccines may not be widely available in some tropical countries such as Colombia. Therefore, control strategies for SI would be highly dependent on strict biosecurity, surveillance, and rapid disease detection [14].

Biosecurity is a set of collective actions and practices that once implemented can reduce the risk of introduction and transmission of diseases into swine farms [23]. Intensive pork production, especially in areas of high densities of farms, rely on strict biosecurity protocols (BP) and good management practices (MP) to minimize the impact of infectious diseases on swine production [24, 25]. Therefore, assessing herd disease risks factors and finding novel strategies to better understand the complex relationships of biosecurity and the epidemiology of infectious diseases in animal productions is highly important [26]. Biosecurity practices are defined as all protocols and management practices implemented in a farm to reduce the risk of introduction or transmission of diseases within and between farms. Biosecurity includes all measures to prevent pathogens from entering a herd (i.e. external biosecurity) and to reduce the spread of pathogens within a herd (i.e. internal biosecurity) [27]. Expected impacts from applying biosecurity measures in livestock farming are improved production characteristics and thus greater profits, better animal welfare, improved immune responses to vaccines and enhanced job satisfaction for farmers [28, 29]. Additionally, higher biosecurity status is linked with a reduction in antimicrobial usage in pig production [27]. Studies have highlighted the importance of biosecurity and its relationships with IAV in pig farms [29]. For example, extensive pig-human contacts, mixing of pigs and other species and suboptimal biosecurity practices adopted by farmers may increase risks for inter-species IAV transmission, adding thereby a threat to pig populations and human public health [30]. Although the benefits and usefulness of biosecurity are known, some studies demonstrated that pig farmers do not implement adequate biosecurity measures [29, 31]. The implementation of BP and MP depends on numerous factors, including the owner perception, the farm production type and the size of the production [31–34]. In this matter, few information is available regarding BP and MP that are implemented in tropical settings such as the Colombian pig farms.

Epidemiological surveys are widely used to evaluate biosecurity and husbandry practices and its relationships with diseases in swine productions [34, 35]. However, the assessment of biosecurity is usually linked to several highly correlated practices [34] making data analysis challenging. Traditionally, descriptive and multivariate methods are used to analyze epidemiological survey data and biosecurity practices from pig farms [36–38]. These statistical methods allow the extraction of fundamental information to understand relationships in large and complex datasets [39]. However, full understanding of the variability and complexity of biosecurity is not a straightforward process, and it is usually not easy to achieve. Applying different and complementary data analysis methods like multiple correspondence analysis (MCA) and hierarchical cluster analysis (HCA) may facilitate the understanding of BP and MP (as a whole and not as single interventions) and their relationships with disease outbreaks in livestock [40].

Therefore, the main goal of our study was to investigate the biosecurity protocols and management practices of Colombian swine farms to identify patterns, relationships, and possible associations with IAV detection at the population level. This study present the findings of a collaborative effort and provide initial insights to better understand the epidemiology of IAV and its association with BP and MP. Our results provide a novel approach of complex survey data from pig farms in Colombia, identifying key elements on farm biosecurity and factors associated with IAV detection in these farms.

## Materials and methods

### Colombian swine industry description

According to the available data for the national swine census published by the Instituto Colombiano Agropecuario “ICA” (Colombian animal health authority) in 2019 there were approximately 240 thousand swine productions, from which one third were backyard systems and about two third were ‘technified’ farms. Of these ‘technified’ farms 95.5% were farms with were farms with inventories of 100 sows or less. Around 6.7 million of pigs were reported for the country, of which 94.9% were pigs from ‘technified’ farms. Overall, more than a half of the pigs (2.9 million) are housed in the most pig dense states; Antioquia (1.7 million), Cundinamarca (0.5 million) and Valle del Cauca (0.7 million) [41].

### Farm selection

This study protocol was presented to the National Association of Pork Producers PorkColombia-Fondo Nacional de la Porcicultura (NAPP) to identify the sampling framework. From census data provided by NAPP we identified a framework of 1397 registered pig farms. After applying our study inclusion criteria [(i) geographic location within the area of study, (ii) willingness to participate, (iii) minimum inventory of 100 sows, and (iv) ease of access] we observed that 364 of these farms fulfilled inclusion criteria. Therefore, a sample size of 187 farms was calculated with 95% confidence level, 5% relative standard error, and 50% proportion for factors [42]. The sample size was proportionally stratified in sample units per each location (state) based on the known distribution of the population. The sampling size was divided into the regions (states) based on the proportion of farms located at each region (census from NAPP), thus we applied a proportional allocation of farms that were evaluated. From the list of candidate farms, 187 of these were randomly selected according to the stratification/allocation approach. A detailed information of the distribution and stratification of the evaluated farms is provided in supplementary material (SI1). Veterinarians from the NAPP overseeing these farms were requested to provide more information about the study to the pig farmers and to invite them to participate in our study by completing an online form. If a farmer expressed interest in the study, we provided the complete study protocol and enrolled into the study after the completion of informed consent.

For the purpose of the study the “Farm” was the unit of interest. Selected farms were first classified according to the size of active breeding herd into: (a) small (100 to 300 sows), (b) medium (301 to 1000 sows), and (c) large (> 1000 sows). Then, farms were classified based on production type into: (a) breeding and nursery farms (production of piglets for sale up to a weight of 22–30 kg), (b) farrow-to-finish farms (pigs are raised from birth to market weight), and (c) genetic core (production only of gilts or boars). Only four genetic core farms were classified in this category and they were all included as farrow-to-finish to avoid their exclusion. Farm location was divided in three regions based on geographical location and upon classification made by the NAPP: (a) Region one (representing 9.51% of the national swine population), comprising the states of Córdoba, Sucre, Bolívar, Magdalena, Cesar, Atlántico, La Guajira, and Norte de Santander; (b) Region two (representing 74.4% of the national swine population), comprising the states of Antioquia, Caldas, Risaralda, Quindío, Valle del Cauca, Cauca, Tolima, and Cundinamarca; and (c) Region three (representing 16.0% of the national swine population), comprising the states of Santander, Boyacá, Arauca, Casanare, Meta, Caquetá, Putumayo, Huila, Chocó, and Nariño. For purposes of this study, we use the term “herd” to mean any group or aggregate of pigs within a farm. Therefore, the individual sites of a multi-site production or the whole aggregate of sites was defined as a herd.

### Farm data and survey information

An epidemiological questionnaire was designed based on previously published data [43]. Questionnaire was presented to the veterinarians from the NNPP. Observations on the content of the questionnaire were taken into account for the design. Questionnaire is available in Spanish upon reasonable request to the corresponding author. The final survey had 80 questions about BP, farm characteristics and MP. Most questions had multiple choice, but sixteen had the option “other” in which the producer could write their answer. Data were collected by personal interviews during the farm visit for sample collection. To avoid bias during interviews, all questions regarding operating procedures were clarified with study personnel prior to self-reporting data collection. All survey data were processed anonymously to protect the confidentiality of the participants. The survey data obtained were coded and transcribed by double entry into an electronic database using Excel (Microsoft, Redmond, Washington, USA).

### IAV sampling, testing and farm characterization

Forty nasal swabs (NS) and eight oral fluids (OF) were collected from piglets between 3 and 12 weeks of age because they are known subpopulations with higher positive rates of IAV detection in epidemically and endemically infected farms [44, 45]. For nasal swabs sample size estimation we assumed a within-farm prevalence of 13% [46], 95% confidence, precision of 10%, and a specificity and sensitivity of 95% for the rRT-PCR test [47]. Specimens were collected at each farm, from groups of at least 30 pigs per pen, in four different pens per farm. Moreover, at convenience, two OF specimens (grouped samples) were also collected from each sampled pen. Group samples have been associated with higher odd ratios of detecting a positive sample by rRT-PCR compared to individual pooled samples [48]. Oral specimen were collected following recommendations previously described [49]. Briefly, ropes were hanged in opposite points of the pen and OF were obtained simultaneously after the ropes were saturated (chewing time of 30 to 60 min). All specimens were transported within 12 to 36 h at 6–10 °C (using gel packs) to the reference lab and stored (-80 °C) until testing.

Viral RNA extraction from specimens (NS and OF) was performed using ZR viral kit (Zymo® research, USA) following manufacturer instructions. Universal molecular detection of IAV matrix gen was carried out by reverse transcriptase real time polymerase chain reaction (rRT-PCR) according to previous methods [47]. Briefly, Fast One Step rRT-PCR kit (Applied Biosystem®, EE.UU.) was used with 0.6 μm of each primer (InfA Forward 5´-GAC CRA TCC TGT CAC CTC TGA C; InfA Reverse 5´-AGG GCA TTY TGG ACA AAK CGT CTA) and 0.2 μm of probe (InfA Probe 5´-GC AGT CCT CGC TCA CTG GGC ACG). Then, 3 uL of extracted RNA were added to 20 uL final reaction volume. Cycling conditions were 50 °C x 5 min, 95 °C x 20 seg, followed of 40 cycles of 95 °C x 15 seg and 60 °C x 1 min. An ABI 7500 Fast (Applied Biosystem®, EE.UU.) thermocycler was used. Specimens from the same farm (same group of pigs and type of sample) were tested in pools (10 NS per pool and 5 OF per pool) to increase testing capacity [50] and if a pool was test positive, then samples composing the pool were tested individually.

### Pilot sample/data collection

The study methods were piloted in six farms prior to verify that the content and interpretations of the questionnaire resulted in reliable and valid measurements, as well as to verify consistency in methodologies for sample/data collection and testing. A detailed information of the pilot results is provided in supplementary material (SI2). Since farmers participation was not as expected and no drawbacks were detected during the pilot, data results from these six farms were included in the study analysis.

### Data analysis

For the purpose of this study a farm was classified as IAV positive if at least one sample (either NS or OF) resulted positive by rRT-PCR. We use descriptive statistics to summarize data. Categorical data were cross-tabulated and quantitative variables were categorized. Tabular methods were used to distribute the number of observations per category. Prior to analysis the number of variables and frequency of missing data was assessed using ‘Amelia’ R-package [51], once missing value was identified, data were inspected to fix any possible data entry or measurement errors. Missing values in the dataset were handled by imputation analysis using a previously reported method using the ‘MIMCA’ procedure in the ‘missMDA’ package for R [52]. A variable was excluded from the analysis if ≥ 20% of missing data was observed, otherwise imputation was conducted on the dataset. Data capturing, data cleaning and summary calculations were done in Excel (Microsoft, Redmond, Washington, USA). Data visualization, processing and analysis was conducted using R software v4.1.2 (R Foundation for Statistical Computing) and RStudio v3.5.0 [53] using different packages. Data analysis followed several stages. Differences on IAV detection on NS and OF was evaluated using Chi-square test (p-value ≤ 0.05).

### Multiple correspondence analysis (MCA)

Categorical data were coded to binary values and then exploratory analysis was conducted using MCA. The MCA helped to identify the most important relationships among variables in the large-complex dataset, by a graphical representation of the similarity between the given observations (Euclidian distance). The MCA was conducted in several stages. A stepwise variable pre-selection approach was followed before final analysis. The large data set was divided into three different subgroups containing similar variables (farm characteristics, management/husbandry practices and biosecurity). These sets of variables were processed separately using MCA. Square cosine criterion (cos2 > 0.2) was used to select the most representative variables during each MCA preprocessing step [54]. Chosen variables were retained and a second MCA was constructed until the final data set was obtained. Inertia (eigenvalue > 0.2) and Cronbach’s alpha score were used to define the number of dimensions to retain in the final MCA [40]. Results of the first two MCA dimensions were plotted to illustrate relationships between variables as MCA principal dimensions. The variables selected from each MCA were then included together in a final MCA, where the most informative variables were retained according to their contribution to the characterization of the farms.

### Hierarchical clustering analysis (HCA)

After exploratory data analysis, result from the MCA were also used as a farm classification method. MCA results were used subsequently to perform an HCA. The clustering analysis with object scores method was used to identify groups of farms sharing similar characteristics within each of the identified dimensions from the MCA, which also aimed to discover an a priori unknown partition among the pig farms. In this sense, we applied a statistical method to classify the farms into clusters based on the level of similarity within and between members of different clusters. Therefore, a relevant clustering trend of the dataset was obtained by HCA. The number of clusters of the HCA model was validated using a partition-based algorithm by the Bayesian information criterion (BIC) [55]. Hypergeometric test (p-value ≤ 0.05) was used to compare the distribution of the variables in the final HCA model, and to identify which subcategories were overrepresented or underrepresented in the characterization of each cluster [56].

### Multivariate regression analysis

After data exploration and univariate analysis of all predictors for IAV detection, we applied a logistic regression analysis using a Generalized Linear model (GLM) by multivariable binomial regressions using logit link to estimate the Odds Ratio (OR) along with their respective 95% confidence intervals (95%CI). Because the unit of analysis was the farm, it was assumed that all samples from pigs in that farm were nested within it. The IAV infection status of the farm was defined as the dependent variable. The building strategy for GLM was conducted in several steps to maximize the control of confounders. In the first step, an identification of a priori variables was made using statistical and non-statistical criteria. Also, variables potentially associated with IAV detection were purposeful selected according to multiple criteria: known scientific or biological evidence of association (see supplementary material SI3), results of the bivariate analysis (χ2 test of independence) performed to identify a possible association (p < 0.05) between the frequency of IAV positive and negative farms in the presence of the variable of interest. In addition, variables that most contributed to the inertia in the MCA solution were also considered. All variables that were plausible for the presence of the virus or that had clinical, epidemiological or biological relevance were considered. In the next step, GLM was built again with all retained variables from previous step. Selection of variables at this step followed a stepwise approach. Model selection was based upon a manual and automatic stepwise removal process (forward and backward) according to their Wald test p-values. The likelihood ratio test was used to identify possible variable contribution to the model. Variables not contributing (p-value > 0.05) were excluded from the model. Confounding was assessed by monitoring coefficients (Δβ) of other variables in the model before and after variable removal. A change in the Δβ was suggestive of possible confounding. Thus, if a change of > 20% on Δβ was observed after any variable exclusion, then the variable was a potential confounding variable and it was returned to the model [57]. This cycle of inclusion/exclusion of variables continued until the best-fitted model was obtained. The best-fitted model was selected based on several model diagnostic criteria, using Pearson’s χ2 test (p < 0.05), Akaike Information Criterion (AIC), the proportion of deviance (%) and the Pseudo-R^2^ coefficient of determination [58]. Additionally, estimation of phi coefficient (phi ≤ 1) was applied to identify overdispersion in the best-fitted model [58]. Variance inflation factor (VIF) was also calculated to assess multicollinearity of variables in the best-fitted model. Plausible interactions terms among the variables in the best-fitted model were evaluated by automatic stepwise process using the “MASS” R package. The likelihood ratio test (p-value ≤ 0.1) and the Tukey’s Honestly Significant Difference (Tukey’s HSD) test were used to identify a significant interaction effect [59, 60]. If the test was not significant (p-value > 0.05), then the interaction term was removed from the main model. The bootstrap method was used to validate the final GLM [61]. Finally, adjusted ORs and their CI95% were also calculated for the best-fitted model.

### Ethics Statement

This study was conducted in accordance with international ethical guidelines and standards for the use of animals in research, under the approved protocol by institutional review board of the National Swine producers Association of Colombia (certificate number 20,111,501). Additionally, informed consent was obtained from all swine farm owners participating in this study. All data were used guaranteeing protection and confidentiality of information. The study followed guidelines of the Strengthening the Reporting of Observational Studies in Epidemiology (STROBE).

## RESULTS

### Farm data collection

Results from 176 (94.11%) farms out of 187 selected farms were evaluated in this study. In total 7,264 NS samples and 1,431 OF specimens were collected and tested. Of these specimens, 247 samples (224 NS; 23 OF) were obtained from six farms during a pilot run (October 2016). Number of samples varied due to technical problems during sample collection in the first farm visited so 47 NS and 3 OF were collected only at this farm. Overall, 7,040 NS samples and 1,408 OF specimens were tested from the participating farms after running the pilot. Data and sample collection started in October 2016 and ended in July 2017. Summary of the distribution and influenza status of the evaluated farms is presented in Table 1. The total sow inventory in the evaluated farms (n = 75,665) represented a 34.8% of the total national inventory of sows (n = 216,837) registered in 2017 for the country. We found 33.5% (59/176) of the farms as positive to IAV. We observed a wide variability on the application of biosecurity, management and husbandry practices in the evaluated farms. A detailed summary of survey data and frequencies of reported data from the farms is shown in Table 2. After dataset exploration, overall missingness was 5%. Missing data ranged from 1% (variable V2-Near (≤ 5 km) to other farms) to 33% (variable V25- Origin of gilts in the last semester). We conducted multiple imputation of variables with missing values below 20%. Three (V25- Origin of gilts in the last semester, V26- Farm of gilt source, V27- Quarantine time for new gilts) variables were excluded from the final multivariate analysis due to missing values above 20%. Distribution of missing values is presented in the supporting information (SI4).

**Table 1:** Location and influenza A virus (IAV) status of 176 swine farms that participated in a biosecurity and husbandry practices survey in Colombia in 2016-17. Nasal swabs (NS) and oral fluids (OF) collected from the surveyed farms were tested for IAV using real time RT-PCR.

Zone code	State	Negative farms	Positive farms	Total farms	IAV Positive samples from NS	IAV Positive samples from OF
1	Atlántico	4	2	6	4	4
	Bolivar	2	0	2	0	0
	Cordoba	1	0	1	0	0
	Magdalena	3	0	3	0	0
	Norte de Santander	1	0	1	0	0
2	Antioquia	45	20	65	84	58
	Caldas	8	2	10	7	2
	Cundinamarca	22	5	27	25	17
	Quindío	2	4	6	20	11
	Risaralda	3	1	4	9	4
	Tolima	3	1	4	0	3
	Valle del Cauca	15	18	33	94	42
3	Boyacá	4	1	5	0	1
	Caquetá	1	0	1	0	0
	Cauca	0	3	3	34	9
	Huila	0	1	1	1	3
	Meta	2	1	3	10	3
	Nariño	1	0	1	0	0
**Total farms**		**117**	**59**	**176**	**288**	**211**

**Table 2:** Results of a survey on biosecurity and husbandry practices of 176 swine farms evaluated in Colombia in 2016-17. Variables and categories included in the study analysis, and their observed frequencies in the farms surveyed. Variables in bold italic were identified as potentially associated with IAV infection status in farms by univariate analysis by Pearson’s χ2 test (p < 0.05)

I. Farm characteristics				
Variable code	**Variable**	**Variable categories**		**Frequency**
***V1***	***Farm location based on regional pig densities***	≥ 10 pigs/km^2^		114 (64.8%)
		< 10 pigs /km^2^		62 (35.2%)
V2	Near (≤ 5 km) to other farms	Yes		87 (49.4%)
		No		88 (50.0%)
		Not response		1 (0.6%)
V3	Farm type	Breeding and nursery		44 (25.0%)
		Farrow-to-finish		128 (72.7%)
		Genetic core		4 (2.3%)
V4	Number of production sites	One site		80 (45.5%)
		Two sites		40 (22.7%)
		More than two sites		51 (28.9%)
		Not response		4 (2.3%)
***V5***	***Farm size***	Small (100 to 300 breeding females)		106 (60.2%)
		Medium (301 to 1000 breeding females)		52 (29.5%)
		Large ((> 1000 breeding females)		14 (8.0%)
		Not response		4 (2.3%)
***V6***	***Total animal inventory***	Less than 2500 pigs		106 (60.2%)
		More than 2500 pigs		70 (39.8%)
II. Biosecurity - Infrastructure				
V7	Floor	Concrete or washable material	169 (96.0%)	
		Other	4 (2.3%)	
		Not response	3 (1.7%)	
V8	Quarantine area independent of production	Yes	109 (61.9%)	
		No	33 (18.8%)	
		Do not have	31 (17.6%)	
		Not response	3 (1.7%)	
V9	Perimeter barrier	Yes	159 (90.3%)	
		No	17 (9.7%)	
V10	Bird nets in swine facilities	Yes	59 (33.5%)	
		No	105 (59.7%)	
		Not response	12 (6.8%)	
III. Biosecurity - Cleaning and disinfection				
V11	Bench or entry system for all personnel	Clothing changing only	5 (2.8%)	
		Shower, clothing and shoes change	116 (65.9%)	
		Clothing and shoes change	43 (24.4%)	
		None	10 (5.7%)	
		Not response	2 (1.1%)	
V12	*Use of bench or entry system is mandatory for all personnel (staff and visitors)*	Yes	137 (77.8%)	
		No	32 (18.2%)	
		Not response	7 (4.0%)	
V13	*Disinfection system for vehicles at entry*	Spray arch	48 (27.3%)	
		Wheel bath	7 (4.0%)	
		Backpack pump sprayer	95 (54.0%)	
		None	15 (8.5%)	
		Not response	11 (6.3%)	
V14	*Cleaning protocol between vehicle usage*	Washing	129 (73.3%)	
		Washing and disinfection	32 (18.2%)	
		None	7 (4.0%)	
		Not response	8 (4.5%)	
V15	*Record vehicle disinfection at entry to the farm and after use*	Yes	129 (73.3%)	
		No	47 (26.7%)	
V16	*Record routine disinfection of facilities*	Yes	115 (65.3%)	
		No	61 (34.7%)	
V17	*Apply disinfection to the facilities after washing*	Yes	164 (93.2%)	
		No	7 (4.0%)	
		Not response	5 (2.8%)	
V18	*Down time of the facility after cleaning and disinfecting*	Two days or less	19 (10.8%)	
		Three days	43 (24.4%)	
		Over four days	89 (50.6%)	
		None	14 (8.0%)	
		Not response	11 (4.5%)	
***V19***	***Quality of water for animal consumption is verified***	Yes	124 (70.5%)	
		No	35 (20.0%)	
		Not response	17 (9.7%)	
***V20***	***After cleaning and disinfecting, the facilities are allowed to dry before use***	Yes	134 (76.1%)	
		No	11 (6.3%)	
		Not response	31 (17.6%)	
IV. Biosecurity - Animal movements				
V21	Mixture of pigs from different origins	Yes	21 (11.9%)	
		No	143 (81.3%)	
		Not response	12 (6.8%)	
V22	Other animals raised within the pig farm	Poultry and cattle	17 (9.7%)	
		Poultry	2 (1.1%)	
		Cattle	91 (51.7%)	
		Horses	8 (4.5%)	
		Sheep / Goats	1 (0.6%)	
		None	57 (32.4%)	
***V23***	***Other animals have access to the swine facilities***	Yes	44 (25.0%)	
		No	119 (67.6%)	
		Not response	13 (7.4%)	
V24	Source of gilts in the last semester	External (from other farms)	79 (46.7%)	
		Internal (from same farm)	80 (45.5%)	
		None	10 (5.7%)	
		Not response	7 (4.0%)	
V25	Origin of gilts in the last semester	Only one source	99 (56.3%)	
		Multiple sources	19 (10.8%)	
		Not response	58 (33.0%)	
V26	Farm of gilt source	Genetic core	106 (60.2%)	
		Commercial farm	16 (9.1%)	
		Imported	3 (1.7%)	
		Other (animal fair - market)	12 (6.8%)	
		Not response	39 (22.2%)	
V27	Quarantine time for new gilts	20 days	17 (9.7%)	
		20 to 30 days	33 (18.8%)	
		More than 30 days	77 (43.8%)	
		Not response	49 (27.8%)	
V28	Wash and disinfects quarantine area between batches	Yes	52 (29.5%)	
		No	123 (69.8%)	
		Not response	1 (0.6%)	
V29	Wash and disinfects gestation barn between groups	Yes	37 (21.0%)	
		No	138 (78.4%)	
		Not response	1 (0.6%)	
***V30***	***Wash and disinfects nursery rooms between groups***	Yes	83 (47.2%)	
		No	92 (52.3%)	
		Not response	1 (0.6%)	
V31	Wash and disinfects areas between groups of growing pigs	Yes	100 (56.8%)	
		No	75 (42.6%)	
		Not response	1 (0.6%)	
V32	Wash and disinfects area between groups of finishing pigs	Yes	72 (40.9%)	
		No	103 (58.5%)	
		Not response	1 (0.6%)	
V. Biosecurity - Transport and personnel				
***V33***	***Independent vehicle to transport animals and feed***	Yes	101 (57.4%)	
		No	21 (11.9%)	
		Not response	14 (8.0%)	
***V34***	***Record of vehicles at entry***	Yes	132 (75.0%)	
		No	37 (21.0%)	
		Not response	7 (4.0%)	
V35	Specialized vehicles to transport animals	Yes	130 (74.0%)	
		No	39 (22.2%)	
		Not response	7 (4.0%)	
V36	Specialized vehicles to transport feed	Yes	121 (73.3%)	
		No	44 (26.7%)	
		Not response	11 (6.3%)	
V37	Type of vehicle to transport animals	Metal bodywork vehicle	75 (42.6%)	
		Wooden bodywork vehicle	92 (52.3%)	
		Other	5 (2.8%)	
		Not response	4 (2.3%)	
V38	Controlled access of visitors to the farm	Yes	162 (92.0%)	
		No	7 (4.0%)	
		Not response	7 (4.0%)	
V39	Workers assigned exclusively to each farm section	Yes	146 (83.0%)	
		No	27 (15.3%)	
		Not response	3 (1.7%)	
V40	Down time for visitors before entering to the facilities	24 h	26 (14.7%)	
		48 h	86 (49.0%)	
		72 h	46 (26.1%)	
		None	13 (7.4%)	
		Not response	5 (2.8%)	
V41	Records of personnel at entry	Yes	129 (73.3%)	
		No	35 (19.8%)	
		Not response	12 (6.8%)	
V42	Non-shared provisions for staff and visitors	Yes	155 (88.1%)	
		No	12 (6.8%)	
		Not response	9 (5.1%)	
V43	Transportation vehicles for exclusive use on the farm	Yes	94 (53.4%)	
		No	48 (27.3%)	
		Not response	34 (19.3%)	
VI. Biosecurity - Health				
***V44***	***Control program for flies***	Yes	162 (92.0%)	
		No	9 (5.1%)	
		Not response	5 (2.8%)	
V45	Rodent control program	Yes	164 (93.2%)	
		No	8 (4.5%)	
		Not response	5 (2.8%)	
***V46***	***Medication and deworming protocols implemented***	Yes	133 (76.0%)	
		No	28 (15.9%)	
		Not response	15 (8.5%)	
V47	Record biosafety programs	Yes	144 (81.8%)	
		No	32 (18.2%)	
***V48***	***Record health care programs***	Yes	127 (72.2%)	
		No	49 (27.8%)	
VII. Management practices				
***V49***	***Ventilation system***	Natural		151 (85.8%)
		Mechanic		3 (1.7%)
		Other		22 (12.5%)
V50	Frequency of technical assistance	Permanent		34 (19.3%)
		Weekly		54 (31.0%)
		Biweekly		25 (14.2%)
		Monthly		52 (29.5%)
		Not response		11 (6.3%)
V51	Type of professional providing technical assistance	Veterinarian		145 (82.4%)
		Animal science		11 (6.3%)
		Agronomist		6 (3.4%)
		Other		3 (1.7%)
		None		6 (3.4%)
		Not response		5 (2.8%)
V52	Frequency of biosecurity training of technicians and operators	Annual		11 (6.3%)
		Biannual		41 (23.3%)
		Quarterly		101 (57.4%)
		None		9 (5.1%)
		Not response		14 (8.0%)
V53	Tail docking in piglets	Yes		169 (96.0%)
		No		18 (10.2%)
		Not response		7 (4.0%)
V54	Breeding system	Natural breeding		7 (4.0%)
		Artificial insemination		151 (85.8%)
		Not response		18 (10.2%)
V55	Some method of castration (chemical or physical)	Yes		41 (23.3%)
		No		127 (72.2%)
		Not response		8 (4.5%)
V56	Type of food supplied	Feed		170 (96.6%)
		Farm-own made mix		3 (1.7%)
		Not response		5 (2.8%)
V57	Feeding system	Manual		124 (70.5%)
		Automatic		37 (21.0%)
		Other		7 (4.0%)
		Not response		8 (4.5%)
VIII. Other supplementary variables				
V58	Geographical location or region	Zone One (Córdoba, Sucre, Bolívar, Magdalena, Cesar, Atlántico, La Guajira, Norte de Santander)		13 (7.38%)
		Zone Two (Antioquia, Caldas, Risaralda, Quindío, Valle del Cauca, Tolima, Cundinamarca)		149 (84.6%)
		Zone Three (Santander, Boyacá, Arauca, Casanare, Meta, Caquetá, Putumayo, Huila, Cauca, Nariño)		14 (7.95%)
V59	Percentage of response to the questionnaire	Answered more than 90% of the questions (High)		148 (84.1%)
		Answered between 70 and 90% of the questions (Medium)		26 (14.8%)
		Answered less than 70% of the questions (Low)		2 (1.1%)
IAV	Influenza virus infection status	Infected farm		59 (33.5%)
		Not infected farm		117 (66.5%)

84% (149/176) of farms were in the same geographical location (zone two) which include the most important pork producing areas in the country, and 64.8% (114/176) of farms were located in a region with pig densities ≥ 10 pigs/km2. Most participating farms were small and farrow-to-finish (61.6% and 72.7% respectively). There was no significant difference in the proportion of herd sizes reported for each farm type (p-value > 0.05). Almost half of the producers interviewed (49.7%; 87/175) reported their farm in close proximity (< 5 km) to other pig farms.

About biosecurity and management practices, we found that 18% (31/173) of farmers reported not having any quarantine area (V8) in place for incoming pigs, compared to 63% (109/173) of farms having quarantine area independent of the production facility. Above a third (46.7%, 79/169) of all producers did not maintain a closed breeding herd (V24), regularly introducing new gilts to the main herd. The majority of producers (83.9%, 99/118) introduced new gilts from one source of origin (V25). Regarding sources of newly introduced gilts in the previous semester (V26), genetic core farms seemed to be an important source for all producers (77.4%, 106/125). The report of rising other livestock animals in addition to pigs (V22) was impressively common among participating farmers (67.6%, 119/176). However, larger farms were less likely to keep other domestic animals than medium and small farms (p-value < 0.05).

Cleaning and disinfection practices and measures related with transport and personnel that were reported by participating farms are summarized in Table 2 (section III). The majority (76.9%, 121/165) of farmers reported using specialized vehicles to transport animals (V36) and about half (53.5%, 92/172) of the farmers reported wooden bodywork vehicle to transport the pigs. The majority (66.2%, 94/142) of the animal transports were of exclusive use in the farm (V43). Washing rather than washing and disinfecting (V14) was the most common (76.8%, 129/168) practice reported for vehicle cleaning after usage. Backpack pump sprayer (V13) was the most common (57.6%, 95/165) disinfection system for vehicles at entry, followed by the spray arch (29.1%, 48/165). In the majority (95.9%, 164/171) of farms a disinfection method was applied after washing of the facilities (V17).

Health practices and husbandry practices that were reported by participating farms are summarized in Table 2 (section VI and VII). Majority of the farmers (> 70%) reported implementation of pest control (V44, V45), deworming (V46), biosafety and health care programs (V47, V48). The majority of farmers (> 80%) reported use of natural ventilation system (V49), manual feeding (V57), artificial insemination (V54), commercial formulated feed (V56), tail docking in piglets (V3) and depend on a veterinarian professional for technical assistance (V51).

After missing data were evaluated using a diagnostic tool from the Amelia package for R software [62]. We observed that 5% of total data collected were missing and only three of the variables (V25 “Origin of gilts in the last semester”; V26 “Farm of gilt source”; V27 “Quarantine time for new gilts”) were found with more than 20% of missing values. The highest percentage of missing data found was 33%. Before further data analysis, missing values were handled using a previously reported multiple imputation method for categorical variables [52]. Additionally, outliers were not identified after dataset inspection.

Results from univariate analysis after dataset imputation indicated that 13 variables (V1, V5, V6, V19, V20, V23, V30, V33, V34, V44, V46, V48, V57) were significative (p-value < 0.05) and thus potentially associated with IAV infectious status. However, after calculating Odds Ratios (OR) only four (V1, V5, V6, V33) of them were significatively associated with higher relative odds of the occurrence of positivity to IAV. Medium size (V5; 300 to 1000 breeding herds) and large size farms (> 1000 breeding herds) had higher odds of being positive to IAV (OR 3.6; IC95% 1.7–7.8 and OR 13.9; IC95% 3.3–84.3) compared to smaller farms. Also, farms having inventories greater than 2500 pigs (V6) had higher odds of being positive to IAV (OR 3.7; IC95% 1.8–7.8) compared to farms with smaller inventories. Farms located in dense pig farming areas (V1; >10 pigs/km^2^) had higher odds of being positive to IAV (OR 2.2; IC95% 1.2–4.9) compared to farms located in less dense pig farming areas. Farms not having an independent vehicle to transport animals and feed (V33) had higher odds of being positive to IAV (OR 3.5; IC95% 1.6–8.5) compared to farms having an independent vehicle.

### IAV testing and characterization

**Fig. 1:**
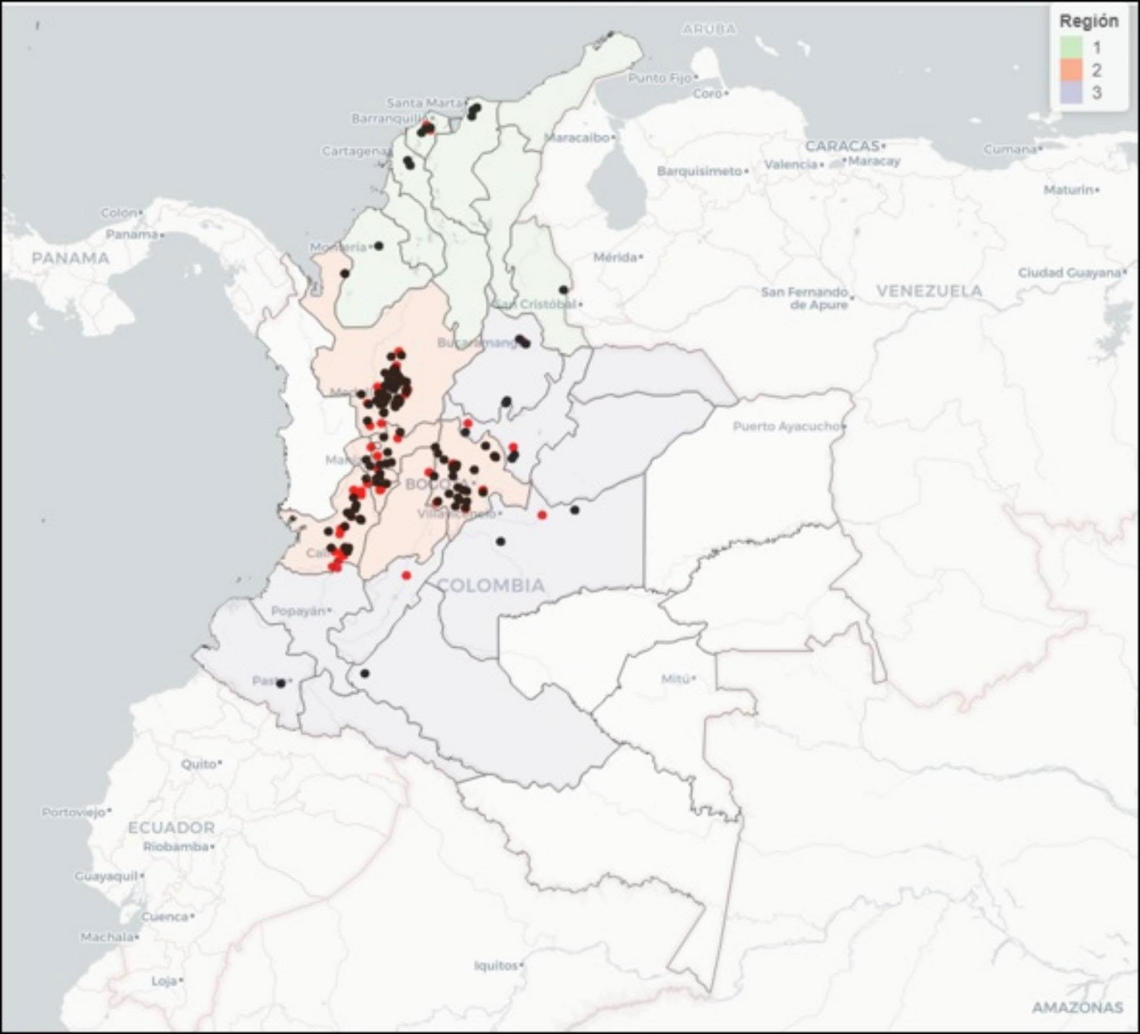
Geographical location of 176 swine farms in Colombia in 2016-17. Farms were surveyed to obtain biosecurity and husbandry practices data. Farms were also tested for Influenza A Virus (IAV) using RT-PCR on nasal swabs and oral fluids. Black dots indicate farms were no IAV positive samples were while red dots indicate farms classified as positive. The location of farms was classified in three zones based on political-administrative division of Colombian states and main pork production regions in the country. Colored zones are light green (zone 1), orange (zone 2) and purple (zone 3)

We tested by rRT-PCR a total of 8,695 specimens (7,264 NS and 1,431 OF) from 176 farms evaluated, and detected IAV in 33.55% (n = 59) of the farms. Positive farms were widely distributed within the main pork producing regions in the country (Table 1; Fig. 1). Furthermore, the prevalence of IAV positive samples at the farm level ranged from 2.08 to 39.58% (data not shown). We also found significantly (p-value < 0.05) less positive NS (3.93%, 275/6,999) than OF (11.03%, 156/1,378), suggesting that OF were more sensitive than NS for IAV detection at the population level (data not shown).

### Multiple xpondence analysis

MCA was used to estimate relationships between 56 qualitative variables that included farm characteristics, herd MP, and BP. After preprocessing steps, 34 out of the 56 variables were selected according to their contribution (cos2 > 0.2) to the inertia, but only 23 variables (Suppl. SI7) in addition to one supplementary variable (region of location) were retained in the final MCA (SI5). Results from the MCA indicated the relationships between the most contributing variables to the inertia that were mainly expressed in 10 principal dimensions (Suppl. SI6). These dimensions accounted for 63% of the variance of the data set, but the final data interpretation was performed taking into account the first two dimensions, which explained about 27.6% of the variance of the data set (cumulative variance). The first principal dimension explained 20.1% of the inertia while the second explained the 7.5%. Variables that most contributed to the inertia in the MCA are showed in the supplementary information (SI7). The final MCA was performed on dataset before and after data imputation. Similar results (26.7% cumulative variance) were obtained for the first two dimensions, however, dataset with imputation was selected to reduce bias and also because the R software package applied performed better on complete datasets.

**Fig. 2:**
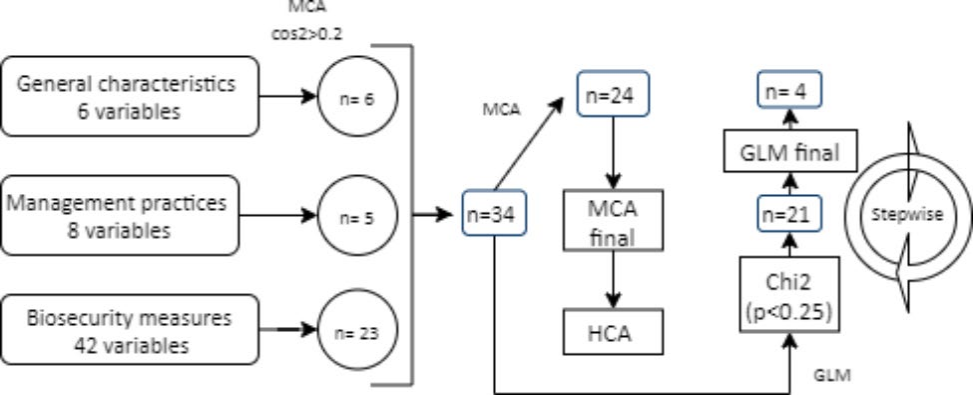
Flowchart describing the processing steps during exploration and analysis of biosecurity and husbandry practices survey data collected of 176 swine farms in Colombia in 2016-17. Univariate and multivariate analysis were performed on survey data after variable selection. Multiple Correspondence Analysis (MCA), Hierarchical cluster Analysis (HCA) and General Linear Modeling (GLM) were performed. The number of variables (n) included at each step of the process is indicated. Variable selection for MCA and HCA was based on the cos2 value, while for GLM was based on p value by Chi square test

### Hierarchical clustering analysis

**Fig. 3:**
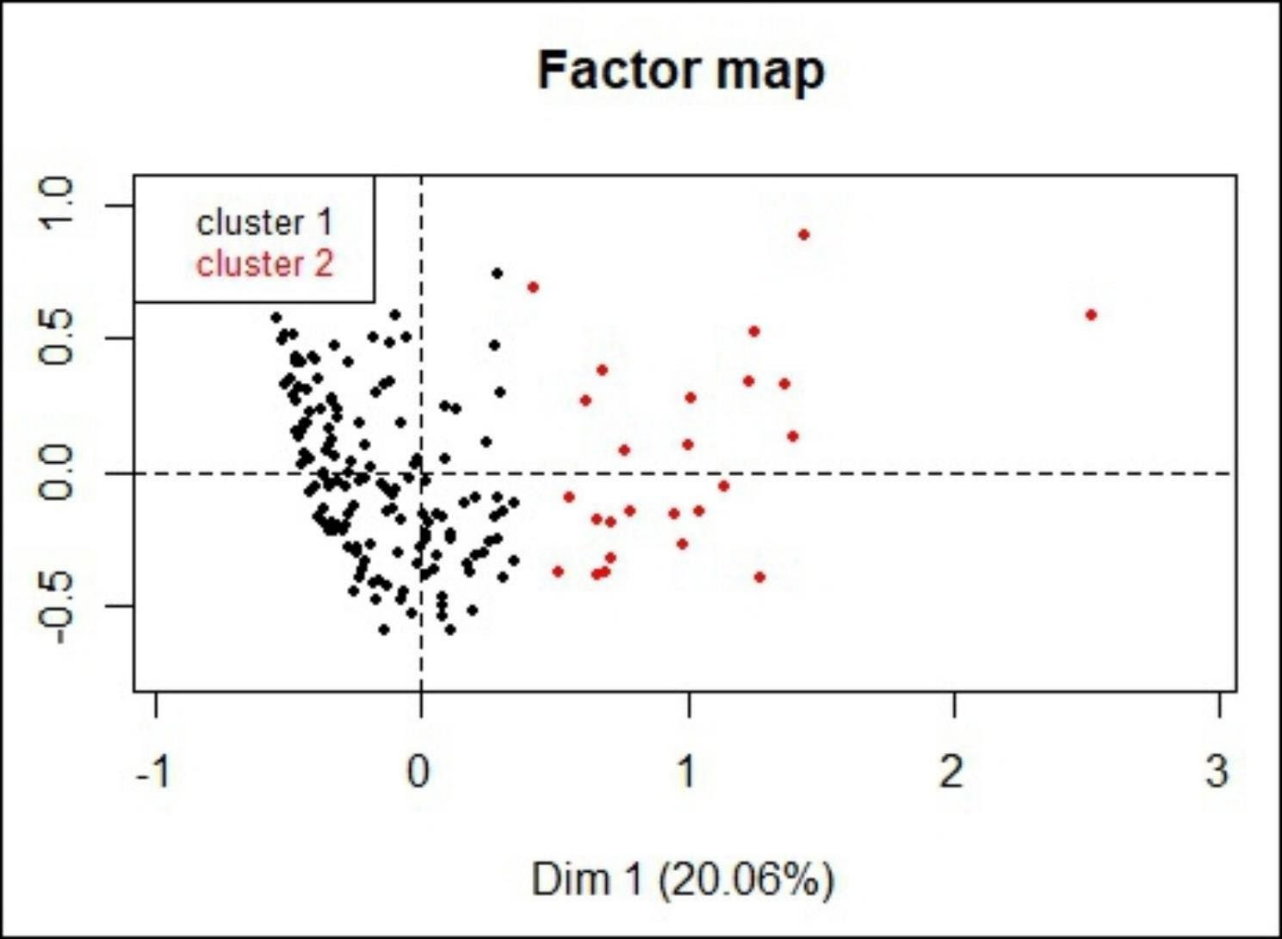
Results of the clustering patterns after multivariate analysis of biosecurity and husbandry practices survey data collected of 176 swine farms in Colombia in 2016-17. Clusters were established based on the similarities between answers to the survey by Multiple Correspondence Analysis; thus, the closer the dots are, the more similar the answers of those farmers were. Colors represents the two different farm clusters. The two perpendicular coordinate axes are referred as dimensions “Dim”. Dim1 (x-axis) and Dim2 (y-axis)

Results from the final MCA solution (23 variables plus one supplementary variable) were subsequently used to perform an agglomerative HCA. The partitioning of the tree clustering from the HCA resulted in a selection of two clusters. Description of the variables used and most influencing variables in the cluster’s selection for the HCA is shown in the supplementary information (SI8-SI9). In total, 85.2% (n = 150) of the farms belonged to cluster 1 while 14.8% (n = 26) of the farms belonged to cluster 2 (Supp. Table 9). Clustering patterns of surveyed data were represented on a factor map (Fig. 3). Model selection was performed according to the BIC criterion and variable selection (using VarSelLCM R package) showed that 78.26% (18/23) of the variables included (plus one supplementary variable) were relevant for clustering. Among the most 10 discriminative biosecurity measures for the clustering were: implementation of records of personnel at farm entry (V41), implementation of record of vehicles at farm entry (V34), application of medication and deworming protocols (V46), mandatory use of bench or “Danish” entry system for personnel (V12), availability of specialized vehicles to transport animals (V35), the type of bench or “Danish” entry system for personnel (V11), workers assigned exclusively to each farm section (V39), allowance of other animals to access to the swine facilities (V23), non-shared provisions for workers and visitors (V42) and implementation of a control program for flies (V44). Hypergeometric test was significant (p < 0.05) and over or under-representation of the data for each category was not observed.

Regarding the main differences of biosecurity measures between farm clusters, we observed that majority of farms in cluster 1 reported shower-in and changing of cloth and shoes as mandatory entry protocol for personnel (112/150; 74.7%) while half of farms in cluster 2 reported only clothing change as the entry protocol for personnel (12/26; 46.2%). Most farms in cluster 1 (99/150; 66%) had a quarantine area located apart from the facility while a third of the farms in cluster 2 had the quarantine area located within the production facility (8/26; 30.8%). Majority of farms in cluster 1 had a disinfection system for vehicles at entry (142/150; 94.7%), while a third of farms in cluster 2 did not disinfect vehicles at entry (8/26; 30.8%). Most farms in cluster 1 had biosafety programs implemented (134/150; 89.3%), while most farms in cluster 2 did not (16/26; 61.5%). Additionally, majority of farms in cluster 1 verified the quality of water for animal consumption (122/150; 81.3%) while half of the farms in cluster 2 did not (13/26; 50%). Most farms in cluster 1 recorded entry of vehicles (134/150; 89.3%) and personnel (135/150; 90%) while farms in cluster 2 did not (21/26 ;84.6% and 22/26 ;84.6% respectively). Majority of farms in cluster 1 used specialized vehicles to transport animals (146/150; 97.3%) or feed (123/150; 82%), while more than half of the farms in cluster 2 did not (20/26; 76.9% and 18/26; 69.2% respectively). Additionally, most farms in cluster 1 had their workers assigned exclusively to each farm Sect. (139/150; 92.7%) while more than half of the farms in cluster 2 did not (17/35; 65.4%). More than half of farms in cluster 1 used independent vehicles to transport feed and animals (102/150; 68%) while more than half of the farms in cluster 2 did not (16/35; 61.5%). Finally, majority of farms in cluster 1 applied a down time above 48 h for visitors before entering to the facilities (122/150; 81.3%) compared to more than a third of farms in cluster 2 that applied a time below 24 h (12/26; 46.2%). A detailed table showing frequencies of variables according to the farm cluster is presented in supplementary material (SI6). Furthermore, the odds of IAV detection were 7.35 times higher in cluster 2 compared to cluster 1 (95% CI: 1.7, 66; p-value < 0.01).

### Farm factors association with IAV infection

Univariate analysis identified a total of 28 variables potentially associated with farm swine influenza status (Table 2). After checking plausibility and biological relevance, we identified 20 of these variables associated with farm swine influenza status (Pearson’s χ2 test; p-value < 0.25). After processing steps of the final logistic regression model, stepwise approach resulted in the selection of 5 potential predictors associated with the detection of IAV in the evaluated farms: “farm size (V5)”, “facilities are allowed to dry after cleaning and disinfecting (V20)”, “location in an area with a high density of pigs in the region (V1)”, “Flies control program (V44)”, “Frequency of training of technicians and operators (V52)”. After assessing interactions among the predictors, stepwise selection approach resulted in 3 potential predictors including an interaction term. The excluded predictors (V44 and V52) were assessed confirming that no confounding effect was presented. Summary of results for logistic regression analysis is presented in Table 3. The main regression model included only these predictors (GLM: iav ~ V5 + V20 + V1 + V5:V1), which were associated with IAV detection (p-value ≤ 0.05). After post-hoc test comparisons, significative contribution was found for all predictors. Adjusted OR for each of the predictors ranged from 0.27 to 34.9 (Table 3). Final main regression model only explained 25.17% of variability observed in the frequency of testing IAV positive attributed to the predictors. From this percentage, 19.52% was caused by partial effects of the model predictors and remaining percentage was related to contribution of complex interrelationships among model predictors (concomitance). Model outliers nor overdispersion was identified.

**Table 3:** Summary of results of logistic regression analysis on Influenza A Virus (IAV) positive detection on survey data collected from 176 swine farms in Colombia in 2016-17. Farms were tested for IAV using rRT-PCR on nasal swabs and oral fluids. Table show results of the final model after controlling potential confounding factors and assessing plausible interactions AIC = 189.92; BIC = 219 Nagelkerke’s R^2^: 0.33 p value = 4.14e-09 Over dispersion coefficient (phi): 1.04 Deviance = 0.269; Concomitance = 5.65%; Main effects sum = 19.52% NSC: Non-Standardized Coefficient; SE: Standard Error; CI: Confidence interval; T: Tolerance; VIF: variance inflation factor; OR: Odds ratio; AIC: Akaike criterion; BIC: Bayesian information criterion; R^2^: adjusted correlation coefficient. Italic bold: statistical significance

Variable	Variable category	NSC		OR	CI 95%		Collinearity	
		β	SE		Lower limit	Upper limit	T	VIF
Intercept		-5.62	1.62	0.003	0.00	0.08	-	-
Farm size (V5)	1001–7500	3.55	1.11	***34.9***	3.92	311.8	0.39	2.54
	301–1000	2.18	0.67	***8.88***	2.38	33.06	0.28	3.50
Facilities are allowed to dry after cleaning and disinfecting (V20)	Yes	3.21	1.55	***24.89***	1.18	524.3	0.97	1.02
Location in an area with a high density of pigs in the region (V1)	> 10 an/km^2^	1.61	0.59	***5.05***	1.56	16.3	0.44	2.25
Farm size (V5): Location in an area with a high density of pigs in the region (V1)	1001–7500: >10an/km2	-1.20	1.25	0.29	0.02	3.4	0.40	2.48
	01-1000: >10an/km2	-1.30	0.76	0.27	0.06	1.2	0.27	3.58

## Discussion

This study assessed the application of farm biosecurity and husbandry practices in Colombia and its association with IAV detection. The study focused firstly on farms with inventories over 100 sows. In this cross-sectional study we used a combination of different methods for analysis of complex survey data to identify and characterize the structure and clustering patterns of Colombian swine farms. Two clustering patterns were found and four variables were associated with IAV detection in these farms. Thus, providing key baseline data to further investigate risk factors of IAV and help to recognize gaps on biosecurity in the Colombian pork industry. Our results also demonstrate the need of more awareness campaigns to reduce undesirable practices in the swine farms in the country. To our knowledge, this is the first study analyzing complex survey epidemiological data from swine farms in Colombia.

Combining multivariate methods such as MCA and HCA, complex survey dataset are reduced to profiles that maximize farm inter-cluster variation and intra-cluster correlation, which allowed us to identify clustering patterns of swine farms and providing key insights on relationships between data. In our study, these combined methods resulted in two clusters of pig farms with different odd of IAV detection. Thus, rising new research questions for future studies to help identify specific gaps on farm biosecurity. Multiple studies have explored risk factors of IAV infections in pig farms [63], however, few studies have focused on analysis of clustering patterns of complex datasets from epidemiological surveys and on establishing their associations with IAV detection at a farm level. In this sense, our results are useful to more realistically understand which potential factors may be affecting the transmission of IAV among the evaluated pig farms.

Pig farms evaluated in this study had in common several characteristics regardless the farm clusters observed. The use of barriers to control access and entry to the farm was common among other practices. However, about a third of the farms in cluster 2 did not have barriers preventing the entry of people, and most of these farms were farrow-to-finish small farms. These results agree with those by other studies which reported that larger farms located in high pig density regions implement higher biosecurity [31]. In our study, most of the larger farms are likely to have a higher technical standard in biosecurity, but risky practices such as mixing of livestock species is still observed.

In our study, we observed a low (27.6%) percentage of the variance explained by the multivariate model, which suggests that the combination of practices implemented by a given farm altogether with the farmer´s behaviors, have a certain degree of randomness and consequently, the farm clustering pattern still contain internal variability. Similar findings are reported in other studies in pig farms [64]. On the other hand, important measures to reduce the risk of disease introduction by visitors and vehicles are not applied on a considerable number of farms [31]. Therefore, to our understanding this is an indication of the complexity surrounding the evaluation of biosecurity, and also acknowledging that compliance of the measures is critical to the analysis of the situation. In our work, compliance was not assessed, and therefore the observed data heterogeneity is probably linked to the different degree of implementation of biosecurity, the expertise of farmers or swine veterinarians, their personalities, their interpretation of biosecurity principles and their access to sources of technical information [31]. Recent studies have shown that compliance of BP varies depending on farmer´s behaviors, work experience and education [65]. Additionally, the increase in the biosecurity standards by pig farmers could be also motivated by the presence of an outbreak of a new disease in the region [66], and that could further difficult the understanding the complexity of this scenario.

While there is a perception of good biosecurity implemented by swine farmers, our results showed that may be a failure in understanding of the biosecurity principles and compliance of protocols. High percentage of farms reported implementation of most measures assessed in the survey, however, among others risky practices such as raising other livestock within the same farm were still reported. Attitudes towards biosecurity can be also influenced by additional factors. Lack of credibility, trust and confusion to the specific recommendations that farmers should follow are some of these affecting factors [31]. In this sense, mixed livestock production is not advisable for several reasons. Potential for cross-species transmission of animal diseases that affect multiple species will be facilitated in areas where concentrations of different animal species co-exist [67]. Our results indicate that small-size pig farmers (< 300 sows) are more likely to engage in this risky practice of mixing livestock. Avian species and poultry may serve as potential reservoirs of IAV within a farm [68]. Phylogenetic studies on IAV (H5N1) viruses have showed transmission from avian species to pigs [69].

Regarding to density of farms, 49.7% of the farms in this study were located close (within a 5 km) to other pig farms. Also, 64.8% of the farms in this study were located in an area of high density of pigs/km^2^. It has been reported that failure in compliance on farm biosecurity in high pig density and mixed farming areas may increase the risk of disease introduction or spread from or to other farms [70]. Also, farms located in areas of high density of pigs have repeatedly been identified as risk factors for IAV seropositivity of farms [59].

Despite the limited sample size and selection criteria of farms included in this study, we believed the trends in the findings of this study generally represent pig farming systems in Colombia. However, very limited assumptions can be made regarding the attitudes and behaviors for biosecurity practices compliance of pig farmers. Nevertheless, at the end of this study, a detailed report was provided to the national swine producer association. Therefore, we believe the provision of our findings, alongside other advisory recommendations will serve as an incentive to review and address the gaps found in biosecurity.

Using logistic regression methods, we found that three variables were associated with higher odds of IAV detection: “location in an area with a high density of pigs in the region (V1)”, “farm size (V5)”, “facilities are allowed to dry after cleaning and disinfecting (V20)”. Several studies have demonstrated that farm size, distance between farms in areas with high densities of pigs, and density of pigs in a specific area are risk factors associated to several viral diseases [71]. In our study, we found that approximately half of the farms were located to less than 5 km from other farms and were located in a pork production area with a local animal density greater than 10 pigs/km^2^. In addition, we observed that some swine farms raised other livestock species making more complex the biosecurity scenario. Previous studies in the United Kingdom, Netherlands and Korea reported great level of difficulty in preventing the spread of viral diseases in dense livestock areas, mainly where a mixture of pigs with other animal species was present [72]. Likewise, a modeling study in China demonstrated how areas of high density of swine productions are highly associated with an increased risk of outbreaks of IAV [73]. Published data have showed that prevalence of IAV is highly associated with high densities of pigs, defined in terms of number of farms and number of pigs [8]. In our study, we found that the frequency of positive farms was significatively higher (p-value = < 0.05) in medium and larger farms, as well as in farms with > 2500 pigs or located in dense (> 10 animals/km^2^) geographical area for pig farming. In this sense, pig density becomes a key element in biosecurity of the farms specially for new facilities. We also found that a positive IAV status increased significantly in larger farms (those with inventories of 300 or more sows), but information about association of farm size to IAV infection in piglets in production systems of Colombia is very limited. Several studies have identified that farm size is a risk factor for IAV infection in pigs in other locations of the world [74]. A metanalysis conducted in 2017, showed that high densities and number of pigs per farm are associated with higher IAV prevalence, suggesting that larger pig farms have higher odds of IAV detection or persistent infections [8]. Other studies revealed that large number of pigs as one influential risk factor for IAV seropositivity in sows and fattening pigs [75], and this factor may also strongly impact on the incidence of subclinical IAV infection [76].

We found an Influenza herd-level prevalence ranging from 2.1 to 39.6% (Median 14.6%) which is lower but similar to findings made for swine farms located in important pork producing regions in the world [43]. It also has been reported that IAV herd-level prevalence may vary over time showing seasonal patterns [43]. We also demonstrated that IAV is actively circulating in piglets and probably established across swine herds in the country. For IAV detection at the farm level we selected piglets between 3 and 12 weeks of age because they act as reservoir for enzootic infections in swine populations [77]. Moreover, piglets play a pivotal role in maintaining IAV endemicity in pig populations. Swine IAVs can endemically persist in farrow-to-finish farms, causing repeated disease outbreaks in pigs around 8 weeks of age [78]. Additionally, different factors contribute to IAV persistence within swine herds including population dynamics of farrow-to-finish farms, immune status of animals and the co-circulation of distinct subtypes [78]. Furthermore, the odds of IAV detection in farms were 7.35 times higher in cluster 2 compared to cluster 1, and cluster 2 was mainly represented by farms with only one production site, therefore these characteristics may also be contributing factors to the endemically persistency of the virus. Therefore, active surveillance should be routinely recommended to better understand the transmission within and between the swine production systems in Colombia. Nevertheless, is important to note that a negative result for IAV in our study does not rule out the presence of the virus in the sampled farms because influenza could be circulating at lower levels or in different aged pigs from which our sample size may have not been able to detect it.

Our findings demonstrated that IAV is widely distributed in Colombian swine populations at the main producing regions for pork industry. The scarce information available regarding surveillance of IAV in the pork industry in Colombia, could be interpreted as little interest on detection of SI in the local industry, thus we hypothesize that conjunction of the lack of active detection of IAV in swine farms, no vaccine availability, and either deficient or not strict compliance of BP could be adding more difficulty on controlling IAV spread within and between pig farms in the country. This highlight that implementation of a vaccination program against SI (guarantying that vaccine strains match circulating viruses in swine) may be highly advised. Vaccination integrated with other BP is one the most effective strategies to reduce IAV transmission and to control spread of SI, even when the disease is persistently endemic in piglets [77].

Controversially, in our study we found that dry after cleaning and disinfection was associated with a higher odd of IAV detection. Given known facts, cleaning and disinfecting are critical parts of all biosecurity programs [79] since the goal of this process is to decrease pathogens load significantly to a point where disease transmission does not occur. However, many other factors may be affecting the persistency of the virus after cleaning and disinfection. For example, air and surfaces in swine barns during outbreaks of IAV can contain different viral loads representing different exposure levels for animals. A study showed that during SI outbreaks, detection of IAV from air was sustained up to 11 days from reported onset representing an exposure hazard to both swine and people [80]. Other experimental studies have demonstrated that IAV transmission is strongly modulated by temperature and humidity [81]. Therefore, it is possible that our finding is confounded by environmental factors such as persistence of airborne viral particles, local temperature, and humidity or that the period time to let dry the surfaces or the cleaning and disinfection process is not properly executed. Another aspect to highlight is the compliance of the measure surveyed. Even that majority of the farmers indicated that after washing and disinfecting they allowed to dry the facilities before use, based on the controversial finding, we may think that compliance to that measure was not completely applied. Social desirability is also a paradigm for measurement bias in surveys. Studies have showed that people can provide answer to surveys based on a normative behavior to appear correct to interviewers [82]. These and some other possible sources of bias could have influenced the results of our study, therefore, our findings should be taken with caution until they can be validated with further studies, in which environmental factors, compliance and other possible affecting variables are assessed.

On the other hand, in our analysis not using an independent vehicle to transport animals and feed was also found to increase the odds of IAV detection. Studies have revealed that feed and other mechanical vectors can be source of transmission of diseases in swine farms [29]. We did not find studies reporting direct evidences of the transmission of IAV by feed or transportation vehicles, however, some findings are provided for other swine viruses. Feed and transport vehicles were linked to transmission of porcine epidemic diarrhea virus [83–85]. Also, transportation of pigs was considered as a potential transmission route for African swine fever virus [86]. An experimental model showed that contaminated trailers can be a source of transmission of porcine reproductive and respiratory syndrome virus [87]. Another study found that some vehicle drivers using his own boots instead of boots supplied by the farm was significantly associated with an increased risk of infection of classical swine fever virus when transporting pigs [88]. But our finding could be biased because we did not investigate the origin and prior movements of the vehicles, and it was not verified compliance of proper disinfection of vehicles between uses. Thus, this finding requires further investigation to clarify.

We acknowledge some limitations of our study. Selecting farms according to some pre-specified rules may have introduced selection bias in the study. Also, the inclusion of farms was based on the voluntary participation of farmers; and thus, selection bias was probably introduced. Lack of clarity and validity of the answers during analysis of survey data may include bias [89], but rigorous design and validation of the questionnaire can help to control biases. In our observational study, prior data collection, a pilot study, including several training sessions and preliminary interviews with farm owners and sample collection and testing were performed to reduce bias. The questionnaire was conducted by six veterinarians from the National Swine Producers Association. Efforts were made to ensure the clarity of the questions during training sessions, however, the interviewers may have unintentionally influenced the responses, so interviewer bias cannot be completely excluded. Most of the questionnaires were closed, excepting those questions that could allow interviewees to provide more details answers or clarifications. Low response rate is another reported disadvantage of surveys [70], then questions with a minimum response rate of 70% are also required to reduce biases during the analyzes [90], however our study significantly exceeded that minimum response rate. Although the overall response rate was high, one cannot neglect the fact that some questions had a response rate under 95% and this could lead to answers non representative of the full study area population, so bias could also be influencing our results. Some studies have suggested that low response rates are probably related to farmers being reluctant to provide information and also because they may think that the information would be used to put pressure on them and that the results would be used against [91]. Also, willingness to share data by farmers may be influenced by the lack of trust. Farmers may have concerns arising from data use, lack of privacy or lack of clarity from the data benefit-sharing [92]. For future studies would be recommended to provide key information to farmers in areas of concerns for including privacy concerns, potential repercussions from results and data benefit sharing.

Additionally, compliance to the measures of biosecurity surveyed in the swine farms was a factor not assessed in our study and may represent a limitation. We acknowledge that farm biosecurity has evolved over time as swine diseases have been better understood, but effectiveness of farm biosecurity depends largely on the compliance by the personnel involved in the production system [65]. Thus, compliance of BP is always a challenging issue when analyzing biosecurity in animal productions. Poor biosecurity compliance has been reported in animal productions [93] and it is frequently related to lack of knowledge or comprehension of the biosecurity principles, but also to human dimensions such as personality and attitudes [94]. Also, the survey data was collected in a cross-sectional study from interviews which may have led to bias towards answers stating measures believed to be applied on farm rather than confirming measures really applied. It is known that perception of a given biosecurity measure apply can be also strongly influenced by multiple factors [32]. When survey respondents were overseeing multiple swine farms limitations on the data analysis was taken into account by analyzing the data only from the sites where the target piglets were sampled for IAV detection.

We cannot rule out influenza infection in the farms that resulted negative in our study, since IAV could be affecting other pigs in the farms different than the groups of animals we studied. Therefore, we may sub-estimating the occurrence of infections in the farms. But investigations have indicated that testing nasal swabs in weaners and nursery pigs is enough for IAV detection at a farm level [44]. Also, to increase the chance of detection for pig farms with low IAV prevalence (< 15%) it is also recommended to use group sampling methods as we did. However, sampling of different age groups is highly advisable to obtain a comprehensive overview on IAV epidemiology on the farm.

Also, the lack of exhaustiveness in the biosafety questions included in the survey may be biasing our findings. Many internal biosafety measures that are relevant in the transmission of SI such as measures such as the movement of piglets between sows, the change of clothes and boots between batches, hand washing between batches, internal circulation of personnel and use of utensils and tools, and handling of sick pigs were not consulted. Also, this cross-sectional study offers a lower degree of scientific evidence compared with other type of studies design. Moreover, there are inherent limitations in terms of methodological issues, generalizability and internal validity when we are dealing with cross-sectional studies. The use of a quality assessment of biosecurity has limitations that should be considered. Finally, this type of study design has limitations on stablishing causal relationships between factors, thus causal relationships should not be inferred from the results presented.

## Conclusion

Livestock raising is an important sector of the Colombian economy and swine production occupy an important place among livestock industries. Despite the great efforts made to increase biosecurity in the pork industry, fails are still noticed as outbreaks of swine diseases are reported in this sector. Because of the above there is an increased need to provide tools for swine veterinary services to identify gaps on biosecurity in these animal productions and data about potential farm factors associated with swine diseases detection. Such approaches should be based on a thorough understanding of farmer’s behaviors, biosecurity and management practices involved the pig production, which are poorly known and highly diverse within the country. To gather such information in a quantifiable manner, a survey approach was developed to better understand the Colombian swine farms, covering biosecurity and husbandry practices, as well as other aspects such as detection of swine influenza virus.

Our study helped to identify gaps on biosecurity and risky practices in the Colombian pig farms evaluated, as well as provided key recommendations to the swine producer association to help control and prevention of IAV spread in these productions. The need of more educational and awareness campaigns for farmers, a better follow-up on implementation of biosecurity as well as its managing in the pig farms, were among others the key findings provided.

Multivariable techniques such as MCA and HCA were useful tools to analyze the complex and large survey data to assess biosecurity in pig farms. These methods allowed to identify profiles and characterize the pig farms based on the farmer’s behaviors, biosecurity and management practices involved the pig production. Also, these farm profiles were further used to estimate the odds of pathogen detection identifying key elements to design appropriate strategies for swine health monitoring and disease control. The present study of the pig production in Colombia increases our knowledge on the different pig farm characteristics, traditional management/husbandry practices and biosecurity protocols applied, which also describe the structure of the main swine production systems in the country. The analysis of the survey data allowed to identify biosecurity gaps and risky behaviors or risky farm profiles, which could be critical control points across the production chain where to implement mitigation measures. Also, our study revealed and highlighted patterns of farms possible associated with higher odds of IAV detection in pigs. Thus, our survey approach for data collection and analysis can be a promising tool to assess biosecurity in swine farms and to identify factors associated with detection of swine pathogens. The main recommendations from this work are to improve communication strategies to and between swine farmers to enhance biosecurity in the country.

## Electronic supplementary material

Below is the link to the electronic supplementary material.

Supplementary Material 1

## Data Availability

The datasets and materials used and/or analyzed during the current study are available from the corresponding author on reasonable request.
