# Correction to: Farm management practices, biosecurity and influenza a virus detection in swine farms: a comprehensive study in Colombia

**DOI:** 10.1186/s40813-022-00288-5

**Published:** 2022-11-01

**Authors:** Karl Ciuoderis-Aponte, Andres Diaz, Carlos Muskus, Mario Peña, Juan Hernández-Ortiz, Jorge Osorio

**Affiliations:** 1 Universidad Nacional de Colombia sede Medellín. Consortium Colombia Wisconsin One Health, Cra75#61-85, 050034 Medellín, Colombia; 2 Pig Improvement Company, Querato, Mexico; 3 Programa de Estudio y Control de Enfermedades Tropicales- PECET, Universidad de Antioquia, Medellín, Colombia; 4 Asociación Porkcolombia - Fondo nacional de la porcicultura, Bogotá, Colombia; 5 Department of Pathobiological sciences, University of Wisconsin Madison. Consortium Colombia Wisconsin One Health, 53706 Madison, USA

**Correction to: Porc Health Manag 8, 42 (2022)**

10.1186/s40813-022-00287-6

Following publication of the original article [1], it was noted that the Given and Family names of Karl Ciuoderis-Aponte, Andres Diaz, Carlos Muskus, Juan Hernández-Ortiz and Jorge Osorio were transposed. Furthermore, affiliation 2 incorrectly listed the city and country as Hendersonville, North Carolina, USA. It should be Querato, Mexico. The correct authorship panel and affiliation is shown in this Correction article and the original article [1] has been updated.
