# Supplementary material for: Farm management practices, biosecurity and influenza a virus detection in swine farms: a comprehensive study in colombia
Source: Porcine Health Manag. 2022 Oct 5;8:42. doi: 10.1186/s40813-022-00287-6 (PMC9532805; doi:10.1186/s40813-022-00287-6)
Supplement: Supplementary file 1 — Supplementary Material 1 [file 40813_2022_287_MOESM1_ESM.docx]

**SI1. Sample size stratification/ allocation and farms distribution.**

| **Place of location of the farms (State-Department)** | **Number of farms registered that fulfilled study inclusion criteria** | | **Number of candidate farms after sample size stratification and allocation** | **Number of farms participating in the study** |
| --- | --- | --- | --- | --- |
|  | **n** | **%** |  |  |
| Antioquia | 139 | 38.2 | 71.4 | 65 |
| Valle del Cauca | 62 | 17.0 | 31.9 | 33 |
| Cundinamarca | 55 | 15.1 | 28.3 | 27 |
| Caldas | 17 | 4.7 | 8.7 | 10 |
| Atlántico | 12 | 3.3 | 6.2 | 6 |
| Cauca | 12 | 3.3 | 6.2 | 3 |
| Boyacá | 10 | 2.7 | 5.1 | 5 |
| Quindío | 10 | 2.7 | 5.1 | 6 |
| Risaralda | 10 | 2.7 | 5.1 | 4 |
| Tolima | 7 | 1.9 | 3.6 | 4 |
| Magdalena | 5 | 1.4 | 2.6 | 3 |
| Meta | 5 | 1.4 | 2.6 | 3 |
| Santander | 5 | 1.4 | 2.6 | 0 |
| Bolívar | 4 | 1.1 | 2.1 | 2 |
| Caquetá | 2 | 0.5 | 1.0 | 1 |
| Choco | 2 | 0.5 | 1.0 | 1 |
| Córdoba | 2 | 0.5 | 1.0 | 1 |
| Huila | 2 | 0.5 | 1.0 | 1 |
| Nariño | 2 | 0.5 | 1.0 | 1 |
| Norte de Santander | 1 | 0.3 | 0.5 | 1 |
| **Total** | **364** | **100** | **187** | **176** |

**SI2. Results from the sample collection and IAV RT-PCR testing during the pilot of the study.**

| **Farm** | **Nasal swabs** | | **Oral Fluid** | |
| --- | --- | --- | --- | --- |
|  | **Positive** | **Total** | **Positive** | **Total** |
| 1 | 0 | 47 | 0 | 3 |
| 2 | 0 | 40 | 0 | 4 |
| 3 | 9 (22.5%) | 40 | 2 (50%) | 4 |
| 4 | 0 | 40 | 0 | 4 |
| 5 | 6 (15%) | 40 | 2 (50%) | 4 |
| 6 | 0 | 40 | 0 | 4 |
| **Total** | **15 (6.1%)** | **247** | **2 (8.7%)** | **23** |

* Number of samples varied in farm 1 due to logistics during initial sample collection.

**SI3. Evidence of scientific criteria used for variable selection in multivariable regression analysis.**

| **Variable** | **Findings** | **References** |
| --- | --- | --- |
| **Farms characteristics** | | |
| Farm size and pig density | Farms with high number of breeding herd were associated with increased anti-SIV antibodies | López-Robles et al., 2012; DOI: 10.1111/j.1865-1682.2011.01250.x |
|  | Large number of pigs per pen in the post-weaning room increased odds for a herd to be IAV positive | Fablet, et al., 2013; DOI:10.1016/j.prevetmed.2013.07.006 |
|  | Close proximity to other pig farms increased odds for a herd to be IAV positive |  |
|  | Higher density of pig herds in the municipality increased odds for a herd to be IAV positive | Maes et al, 2000; DOI: 10.1051/vetres:2000122 |
|  | Higher number of pigs per pen increased the risk of a herd being positive |  |
|  | Farms containing high numbers of pigs per water space were found to have higher odds of IAV positivity | Mastin et al., 2011; DOI: 10.1371/currents.RRN1209 |
|  | Pig or farm density at different geographic levels were factors associated with herd IAV positivity | Suriya et al., 2008; DOI: 10.1111/j.1863-2378.2008.01138.x |
|  | Size of farm and proximity of farm to other pig farms were factors associated with herd IAV positivity |  |
| **Biosecurity** | | |
| Cleaning and disinfecting procedures | Disinfectants were used inappropriately in livestock farms and may poses a risk for virus spread | Kim et al., 2020; DOI: 10.4142/jvs.2020.21.e34 |
|  | Common principles for biosecurity in pig farms | Alarcón et al., 2021; DOI: 10.1186/s40813-020-00181-z |
| Quality of water | IAV are able to persist in the water along with other influencing factors | Dalziel et al., 2016; DOI: 10.1371/journal.pone.0161929 |
| Other animal species in the swine facilities | Inter-species transmission of IAV | Short et al., 2015; 10.1016/j.onehlt.2015.03.001 |
| Transport vehicles | Feed and transport vehicles were linked to transmission of porcine epidemic diarrhea virus | Aubry et al., 2016; J Swine Health Prod;25(2):69-72 |
|  | transportation of pigs was considered as a potential transmission route for African swine fever virus | Hu et al., 2021; DOI: 10.3389/fphy.2021.785885 |
|  | vehicle drivers using his own boots instead of boots supplied by the farm was significantly associated with an increased risk of infection of classical swine fever virus when transporting pigs | Elbers et al., 2001; DOI: 10.1136/vr.149.13.377 |
| Accessing to the farm | Uncontrolled entrance to the farm was associated with IAV seroprevalence | Simon-Grifé et al., 2011; DOI: 10.1016/j.vetmic.2010.10.015 |
| Insect control programs | IAV has been isolated in blow flies collected in infected poultry farms which highlights the potential role of insects as mechanical vectors. | Sawabe et al., 2006; PMID: 16896143 |
| Medication and deworming protocols | Common principles for biosecurity in pig farms | Alarcón et al., 2021; DOI: 10.1186/s40813-020-00181-z |
| Implementation of health care programs | Common principles for biosecurity in pig farms | Alarcón et al., 2021; DOI: 10.1186/s40813-020-00181-z |
| **Management practices** | | |
| Ventilation system | Controlling of ventilation and other factors (temperature) increased odds for a herd to be IAV positive | Fablet, et al., 2013; DOI:10.1016/j.prevetmed.2013.07.006 |

**SI4. Missing values among surveyed variables. Map obtained using Amelia package for R.**


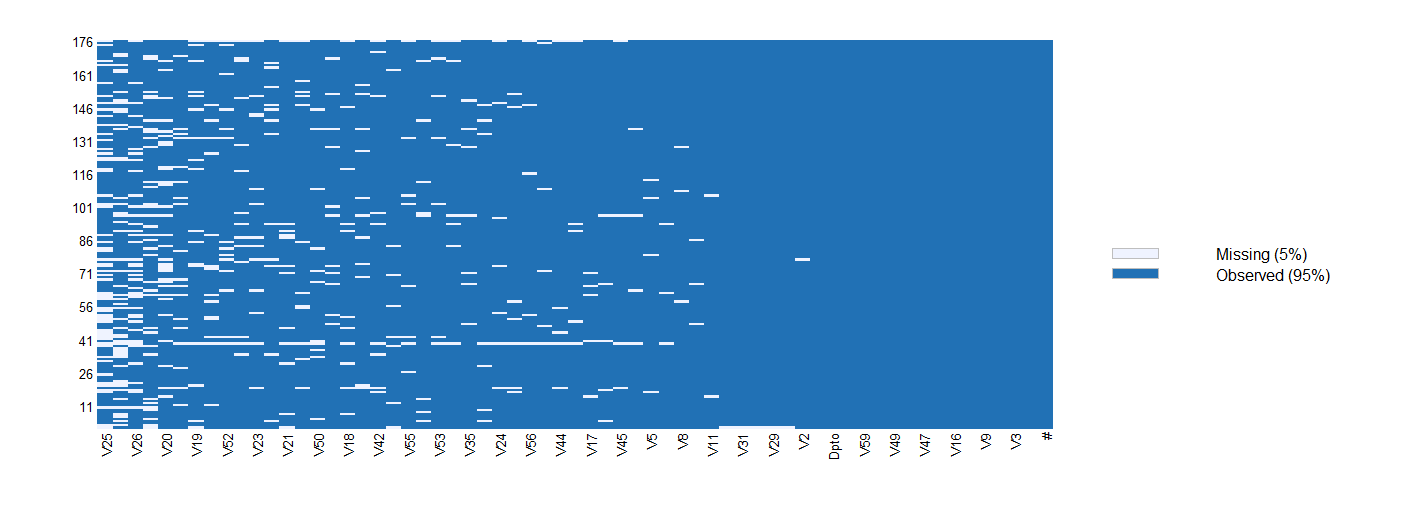


**SI5.** **Distances between categories of the most informative variables for final Multiple Correspondence Analysis (MCA) of biosecurity and husbandry practices survey data collected of 176 swine farms in Colombia in 2016-17.** The two axes in the plot represent dimension 1 (Dim1, x-axis) and 2 (Dim2 y-axis) of the final MCA. The coordinates of each variable correspond to their eigenvalues. The percentages of each axis for Dim1 (20.5%) and Dim2 (7.7%) correspond to the retained inertia for each dimension illustrated. Variables close to each other in the same quadrants are indicative of stronger associations. The top 20 contributing variables are showed. Variable acronyms and their respective categories are specified in suppl. Table 6 (SI6).


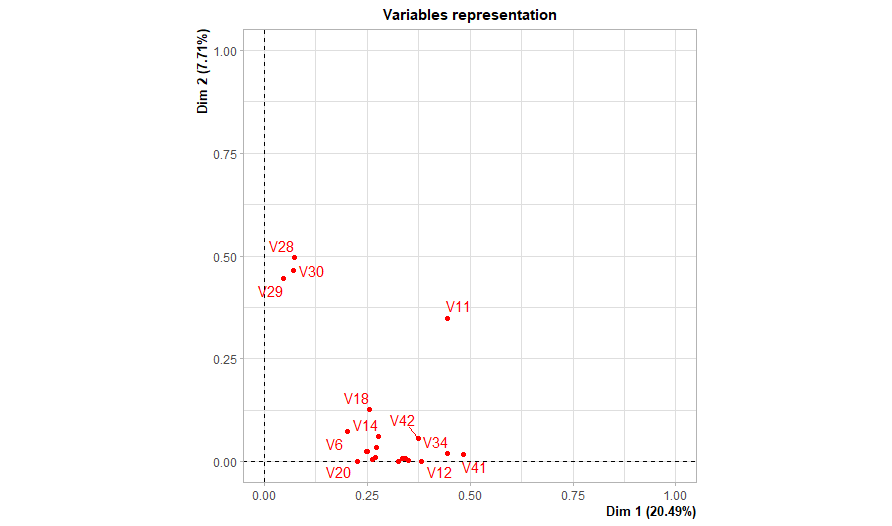
**SI6.** Bar plot of the results after Multiple Correspondence Analysis of biosecurity and husbandry practices survey data collected of 176 swine farms in Colombia in 2016-17. The x axis indicates the principal dimensions identified and the y axis represents the percentage of inertia explained by each dimension.


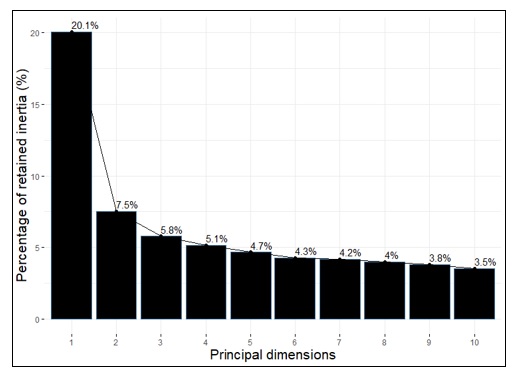


**SI7**. Variables (names and meanings) included in Multiple Correspondence Analysis computed from survey data (biosecurity and husbandry practices) collected on 176 swine farms in Colombia in 2016-17.

| Variable name | Variable code | Variable description |
| --- | --- | --- |
| Total animal inventory | V6 | Total number of pigs (piglets, breeding sows, hogs and growing-finishing) housed in the farm |
| Access system for personnel entry | V11 | “Physical barrier” required by visitors and workers before entering to the farm |
| Use of access system is mandatory | V12 | Describes whether or not an access protocol was enforced to visitors and workers before entering to the farm |
| Cleaning protocol between vehicle usage | V14 | Cleaning method applied to vehicles after usage |
| Apply disinfection to the facilities after washing | V17 | Describes whether or not disinfection was applied to the facilities after washing |
| Down time of the facility after cleaning and disinfecting | V18 | Number of days waited before using again the facility after cleaning and disinfection |
| After cleaning and disinfecting, the facilities are allowed to dry before use | V20 | Describes whether or not the facility was led to dry before next usage |
| Other animals have access to the swine facilities | V23 | Describes which livestock animals have also access to the swine facilities |
| Wash and disinfects quarantine area between batches | V28 | Describes whether or not quarantine area was washed and disinfected between batches |
| Wash and disinfects gestation barn between groups | V29 | Describes whether or not gestation area was washed and disinfected between batches |
| Wash and disinfects nursery rooms between groups | V30 | Describes whether or not nursery area was washed and disinfected between batches |
| Record entry of vehicles | V34 | Describes whether or not a registry of vehicles that have entered to the farm was kept |
| Specialized vehicles to transport animals | V35 | Describes whether or not a specialized type of vehicles was used to transport animals |
| Specialized vehicles to transport feed | V36 | Describes whether or not a specialized type of vehicle was used to transport feed |
| Exclusive personnel to each farm section | V39 | Describes whether or not the farm workers had exclusive job assignation to a farm section |
| Down time for visitors before entering to the facilities | V40 | Number of days of isolation enforced for visitors when they were recently coming from another pig farm |
| Records entry of personnel | V41 | Describes whether or not a registry of people that have entered to the farm was kept |
| Exclusive provision for staff and visitors | V42 | Describes whether or not people had to share with others their protective equipment (boots, work clothes, etc) while working or visiting the farm |
| Flies control program | V44 | Describes whether or not the farm had an active control program for flies |
| Medication and deworming protocols | V46 | Describes whether or not the farm had routine medication and deworming protocols in place |
| Breeding system | V54 | Type of breeding system used |
| Influenza virus infection status of the farm | IAV | Influenza virus infection status of the farm based on RT-PCR testing results |
| State - Supplementary variable | region | Zone where the farm was located based on the political-administrate division of the country |

**SI8.** Description of the most influencing variables in the selection of clusters of a Hierarchical Cluster Analysis of biosecurity and husbandry practices of 176 swine farms surveyed in Colombia in 2016-17. Results from R software using VarSelLCM package.

| **Variable name** | **Discriminative power** | | |
| --- | --- | --- | --- |
|  | **Total value** | **%** | **% Accumulated** |
| V41 Records entry of personnel | 38.45 | 14.92 | 14.92 |
| V34 Record entry of vehicles | 34.33 | 13.32 | 28.24 |
| V46 Medication and deworming protocols | 20.26 | 7.86 | 36.10 |
| V12 Use of access system is mandatory | 18.24 | 7.08 | 43.18 |
| V35 Specialized vehicles to transport animals | 15.05 | 5.84 | 49.02 |
| V11 Access system for personnel entry | 14.88 | 5.77 | 54.79 |
| V39 Exclusive personnel to each farm section | 14.27 | 5.54 | 60.33 |
| V23 Other animals have access to the swine facilities | 14.13 | 5.48 | 65.81 |
| V6 Total animal inventory | 12.25 | 4.75 | 70.56 |
| V42 Exclusive provision for staff and visitors | 11.51 | 4.47 | 75.03 |
| V44 Flies control program | 11.51 | 4.47 | 79.49 |
| V36 Specialized vehicles to transport feed | 10.55 | 4.09 | 83.59 |
| V58. Region of location | 6.67 | 2.59 | 86.18 |
| V54 Breeding system | 6.29 | 2.44 | 88.62 |
| iav | 6.23 | 2.42 | 91.04 |
| V14 Cleaning protocol between vehicle usage | 6.18 | 2.40 | 93.43 |
| V20 After cleaning and disinfecting the facilities are allowed to dry before use | 5.45 | 2.12 | 95.55 |
| V40 Down time for visitors before entering to the facilities | 5.03 | 1.95 | 97.50 |

**SI9.** Description of the most influencing variables for clustering analysis of 176 swine farms surveyed in Colombia in 2016-17.

|  | | | **Cluster 1 n=150; 85.2%** | | **Cluster 2 n=26; 14.8%** | |
| --- | --- | --- | --- | --- | --- | --- |
| **Variable code** | **Variable** | **Variable categories** | tot | % | tot | % |
| V1 | Farm location based on regional pig densities | ≥10 pigs/km^2^ | 104 | 69.3 | 10 | 38.5 |
|  |  | <10 pigs /km^2^ | 46 | 30.7 | 16 | 61.5 |
| V2 | Near (≤5 km) to other farms | Yes | 77 | 51.3 | 11 | 42.3 |
|  |  | No | 73 | 48.7 | 15 | 57.7 |
| V3 | Farm type | Breeding and nursery | 37 | 24.7 | 7 | 26.9 |
|  |  | Farrow-to-finish | 109 | 72.7 | 19 | 73.1 |
|  |  | Genetic core | 4 | 2.7 | 0 | 0.0 |
| V4 | Number of production sites | One site | 66 | 44.0 | 17 | 65.4 |
|  |  | Two sites | 35 | 23.3 | 6 | 23.1 |
|  |  | More than two sites | 49 | 32.7 | 3 | 11.5 |
| V5 | Farm size | Small (100 to 300 breeding females) | 85 | 56.7 | 23 | 88.5 |
|  |  | Medium (301 to 1000 breeding females) | 13 | 8.7 | 1 | 3.8 |
|  |  | Large ((>1000 breeding females) | 52 | 34.7 | 2 | 7.7 |
| V6 | Total animal inventory | Less than 2500 pigs | 81 | 54.0 | 25 | 96.2 |
|  |  | More than 2500 pigs | 69 | 46.0 | 1 | 3.8 |
| V7 | Floor | Concrete or washable material | 147 | 98.0 | 25 | 96.2 |
|  |  | Other | 3 | 2.0 | 1 | 3.8 |
| V8 | Quarantine area independent of production | Yes | 99 | 66.0 | 12 | 46.2 |
|  |  | No | 25 | 16.7 | 8 | 30.8 |
|  |  | Do not have | 26 | 17.3 | 6 | 23.1 |
| V9 | Perimeter barrier | Yes | 138 | 92.0 | 21 | 80.8 |
|  |  | No | 12 | 8.0 | 5 | 19.2 |
| V10 | Bird nets in swine facilities | Yes | 55 | 36.7 | 6 | 23.1 |
|  |  | No | 95 | 63.3 | 20 | 76.9 |
| V11 | Access system for all personnel (visitors and staff) at entry | Clothing changing only | 31 | 20.7 | 12 | 46.2 |
|  |  | Shower, clothing and shoes change | 112 | 74.7 | 5 | 19.2 |
|  |  | Clothing and shoes change | 4 | 2.7 | 1 | 3.8 |
|  |  | None | 3 | 2.0 | 8 | 30.8 |
| V12 | Use of access entry system is mandatory for all personnel (visitors and staff) | Yes | 135 | 90.0 | 8 | 30.8 |
|  |  | No | 15 | 10.0 | 18 | 69.2 |
| V13 | Disinfection system for vehicles at entry | Spray arch | 49 | 32.7 | 3 | 11.5 |
|  |  | Wheel bath | 7 | 4.7 | 0 | 0.0 |
|  |  | Backpack pump sprayer | 86 | 57.3 | 15 | 57.7 |
|  |  | None | 8 | 5.3 | 8 | 30.8 |
| V14 | Cleaning protocol between vehicle usage | Washing | 119 | 79.3 | 18 | 69.2 |
|  |  | Washing and disinfection | 30 | 20.0 | 2 | 7.7 |
|  |  | None | 1 | 0.7 | 6 | 23.1 |
| V15 | Record vehicle disinfection | Yes | 121 | 80.7 | 26 | 100.0 |
|  |  | No | 29 | 19.3 | 18 | 69.2 |
| V16 | Record disinfection of facilities | Yes | 107 | 71.3 | 8 | 30.8 |
|  |  | No | 43 | 28.7 | 18 | 69.2 |
| V17 | Apply disinfection to the facilities after washing | Yes | 148 | 98.7 | 21 | 80.8 |
|  |  | No | 2 | 1.3 | 5 | 19.2 |
| V18 | Down time of the facility after cleaning and disinfecting | Two days or less | 19 | 12.7 | 2 | 7.7 |
|  |  | Three days | 40 | 26.7 | 4 | 15.4 |
|  |  | Over four days | 82 | 54.7 | 11 | 42.3 |
|  |  | None | 9 | 6.0 | 9 | 34.6 |
| V19 | Quality of water for animal consumption is verified | Yes | 122 | 81.3 | 13 | 50.0 |
|  |  | No | 28 | 18.7 | 13 | 50.0 |
| V20 | After cleaning and disinfecting, the facilities are allowed to dry before use | Yes | 144 | 96.0 | 17 | 65.4 |
|  |  | No | 6 | 4.0 | 9 | 34.6 |
| V21 | Mixture of pigs from different origins | Yes | 18 | 12.0 | 4 | 15.4 |
|  |  | No | 132 | 88.0 | 22 | 84.6 |
| V22 | Other animals raised within the pig farm | Poultry and cattle | 11 | 7.3 | 6 | 23.1 |
|  |  | Poultry | 1 | 0.7 | 1 | 3.8 |
|  |  | Cattle | 85 | 56.7 | 6 | 23.1 |
|  |  | Sheep / Goats/ Horses | 6 | 4.0 | 3 | 11.5 |
|  |  | None | 47 | 31.3 | 10 | 38.5 |
| V23 | Other animals have access to the swine facilities | Yes | 31 | 20.7 | 20 | 76.9 |
|  |  | No | 119 | 79.3 | 6 | 23.1 |
| V24 | Source of gilts in the last semester | External (from other farms) | 73 | 48.7 | 9 | 34.6 |
|  |  | Internal (from same farm) | 70 | 46.7 | 14 | 53.8 |
|  |  | None | 7 | 4.7 | 3 | 11.5 |
| V25 | Origin of gilts in the last semester | Only one source | 133 | 88.7 | 16 | 61.5 |
|  |  | Multiple sources | 17 | 11.3 | 10 | 38.5 |
| V26 | Farm of gilt source | Genetic core | 123 | 82.0 | 13 | 50.0 |
|  |  | Commercial farm | 14 | 9.3 | 9 | 34.6 |
|  |  | Imported | 3 | 2.0 | 0 | 0.0 |
|  |  | Other (animal fair - market) | 10 | 6.7 | 4 | 15.4 |
| V27 | Quarantine time for new gilts | 20 days | 17 | 11.3 | 3 | 11.5 |
|  |  | 20 to 30 days | 43 | 28.7 | 8 | 30.8 |
|  |  | More than 30 days | 90 | 60.0 | 15 | 57.7 |
| V28 | Wash and disinfects quarantine area between batches | Yes | 49 | 32.7 | 4 | 15.4 |
|  |  | No | 101 | 67.3 | 22 | 84.6 |
| V29 | Wash and disinfects gestation barn between groups | Yes | 34 | 22.7 | 3 | 11.5 |
|  |  | No | 116 | 77.3 | 23 | 88.5 |
| V30 | Wash and disinfects nursery rooms between groups | Yes | 76 | 50.7 | 8 | 30.8 |
|  |  | No | 74 | 49.3 | 18 | 69.2 |
| V31 | Wash and disinfects areas between groups of growing pigs | Yes | 93 | 62.0 | 8 | 30.8 |
|  |  | No | 57 | 38.0 | 18 | 69.2 |
| V32 | Wash and disinfects area between groups of finishing pigs | Yes | 69 | 46.0 | 4 | 15.4 |
|  |  | No | 81 | 54.0 | 22 | 84.6 |
| V33 | Independent vehicle to transport animals and feed | Yes | 102 | 68.0 | 10 | 38.5 |
|  |  | No | 48 | 32.0 | 16 | 61.5 |
| V34 | Record of vehicles at entry | Yes | 134 | 89.3 | 5 | 19.2 |
|  |  | No | 16 | 10.7 | 21 | 80.8 |
| V35 | Specialized vehicles to transport animals | Yes | 129 | 86.0 | 6 | 23.1 |
|  |  | No | 21 | 14.0 | 20 | 76.9 |
| V36 | Specialized vehicles to transport feed | Yes | 123 | 82.0 | 8 | 30.8 |
|  |  | No | 27 | 18.0 | 18 | 69.2 |
| V37 | Type of vehicle to transport animals | Metal bodywork vehicle | 72 | 48.0 | 4 | 15.4 |
|  |  | Wooden bodywork vehicle | 74 | 49.3 | 21 | 80.8 |
|  |  | Other | 4 | 2.7 | 1 | 3.8 |
| V38 | Controlled access of visitors at entry | Yes | 147 | 98.0 | 21 | 80.8 |
|  |  | No | 3 | 2.0 | 5 | 19.2 |
| V39 | Workers assigned exclusively to each farm section | Yes | 139 | 92.7 | 9 | 34.6 |
|  |  | No | 11 | 7.3 | 17 | 65.4 |
| V40 | Down time for visitors before entering to the facilities | 24 hours | 22 | 14.7 | 4 | 15.4 |
|  |  | 48 hours | 79 | 52.7 | 11 | 42.3 |
|  |  | 72 hours | 43 | 28.7 | 3 | 11.5 |
|  |  | None | 6 | 4.0 | 8 | 30.8 |
| V41 | Records of personnel at entry | Yes | 135 | 90.0 | 4 | 15.4 |
|  |  | No | 15 | 10.0 | 22 | 84.6 |
| V42 | Exclusive provision for staff and visitors | Yes | 148 | 98.7 | 16 | 61.5 |
|  |  | No | 2 | 1.3 | 10 | 38.5 |
| V43 | Transportation vehicles for exclusive use on the farm | Yes | 105 | 70.0 | 9 | 34.6 |
|  |  | No | 45 | 30.0 | 17 | 65.4 |
| V44 | Flies control program | Yes | 149 | 99.3 | 17 | 65.4 |
|  |  | No | 1 | 0.7 | 9 | 34.6 |
| V45 | Rodent control program | Yes | 148 | 98.7 | 19 | 73.1 |
|  |  | No | 2 | 1.3 | 7 | 26.9 |
| V46 | Medication and deworming protocols | Yes | 134 | 89.3 | 8 | 30.8 |
|  |  | No | 16 | 10.7 | 18 | 69.2 |
| V47 | Record biosafety programs | Yes | 134 | 89.3 | 10 | 38.5 |
|  |  | No | 16 | 10.7 | 16 | 61.5 |
| V48 | Record health care programs | Yes | 120 | 80.0 | 7 | 26.9 |
|  |  | No | 30 | 20.0 | 19 | 73.1 |
| V49 | Ventilation system | Natural | 126 | 84.0 | 25 | 96.2 |
|  |  | Mechanic | 3 | 2.0 | 0 | 0.0 |
|  |  | Other | 21 | 14.0 | 1 | 3.8 |
| V50 | Frequency of technical assistance | Permanent | 30 | 20.0 | 5 | 19.2 |
|  |  | Weekly | 52 | 34.7 | 3 | 11.5 |
|  |  | Biweekly | 22 | 14.7 | 4 | 15.4 |
|  |  | Monthly | 46 | 30.7 | 14 | 53.8 |
| V51 | Type of professional providing technical assistance | Veterinarian | 131 | 87.3 | 19 | 73.1 |
|  |  | Animal science | 9 | 6.0 | 2 | 7.7 |
|  |  | Agronomist | 5 | 3.3 | 1 | 3.8 |
|  |  | Other | 3 | 2.0 | 0 | 0.0 |
|  |  | None | 2 | 1.3 | 4 | 15.4 |
| V52 | Frequency of biosecurity training of technicians and operators | Annual | 5 | 3.3 | 7 | 26.9 |
|  |  | Biannual | 38 | 25.3 | 6 | 23.1 |
|  |  | Quarterly | 102 | 68.0 | 7 | 26.9 |
|  |  | None | 5 | 3.3 | 6 | 23.1 |
| V53 | Tail docking in piglets | Yes | 134 | 89.3 | 24 | 92.3 |
|  |  | No | 16 | 10.7 | 2 | 7.7 |
| V54 | Breeding system | Natural breeding | 2 | 1.3 | 7 | 26.9 |
|  |  | Artificial insemination | 148 | 98.7 | 19 | 73.1 |
| V55 | Some method of castration (chemical or physical) | Yes | 30 | 20.0 | 14 | 53.8 |
|  |  | No | 120 | 80.0 | 12 | 46.2 |
| V56 | Type of food supplied | Feed | 147 | 98.0 | 26 | 100.0 |
|  |  | Farm-own made mix | 3 | 2.0 | 0 | 0.0 |
| V57 | Feeding system | Manual | 107 | 71.3 | 24 | 92.3 |
|  |  | Automatic | 36 | 24.0 | 1 | 3.8 |
|  |  | Other | 7 | 4.7 | 1 | 3.8 |
| V58 | Geographical location or region | Zone One (Córdoba, Sucre, Bolívar, Magdalena, Cesar, Atlántico, La Guajira, Norte de Santander) | 4 | 2.7 | 9 | 34.6 |
|  |  | Zone Two (Antioquia, Caldas, Risaralda, Quindío, Valle del Cauca, Tolima, Cundinamarca) | 136 | 90.7 | 13 | 50.0 |
|  |  | Zone Three (Santander, Boyacá, Arauca, Casanare, Meta, Caquetá, Putumayo, Huila, Cauca, Nariño) | 10 | 6.7 | 4 | 15.4 |
| V59 | Percentage of response to the questionnaire | Answered more than 90% of the questions (High) | 130 | 86.7 | 18 | 69.2 |
|  |  | Answered between 70 and 90% of the questions (Medium) | 20 | 13.3 | 6 | 23.1 |
|  |  | Answered less than 70% of the questions (Low) | 0 | 0.0 | 2 | 7.7 |
| IAV | Influenza virus status | Infected farm | 57 | 38.0 | 2 | 7.7 |
|  |  | Not infected farm | 93 | 62.0 | 24 | 92.3 |
